# Discovery and evaluation of novel synthetic 5-alkyl-4-oxo-4,5-dihydro-[1,2,4]triazolo[4,3-a]quinoxaline-1-carbox-amide derivatives as anti-inflammatory agents

**DOI:** 10.1080/14756366.2019.1680658

**Published:** 2019-11-11

**Authors:** Qing-Kun Shen, Guo-Hua Gong, Gao- Li, Mei- Jin, Li-Hua Cao, Zhe-Shan Quan

**Affiliations:** a Key Laboratory of Natural Resources and Functional Molecules of the Changbai Mountain, Affiliated Ministry of Education, College of Pharmacy, Yanbian University, Yanji, China; b Inner Mongolia Autonomous Region Key Laboratory of Mongolian Medicine Pharmacology for Cardio-Cerebral Vascular System, Tongliao, China; c Affiliated Hospital of Inner Mongolia University for Nationalities, Tongliao, China; d Department of Central Laboratory, Yanbian University Hospital, Yanji, China; e College of Medical, Yanbian University, Yanji, China

**Keywords:** Synthesis, anti-inflammatory activity, NO, MAPKs

## Abstract

To develop novel anti-inflammatory agents, a series of 5-alkyl-4-oxo-4,5-dihydro-[1, 2, 4]triazolo[4,3-a]quinoxaline-1-carboxamide derivatives were designed, synthesised, and evaluated for anti-inflammatory effects using RAW264.7 cells. Structures of the synthesised compounds were determined using ^1^H NMR, ^13 ^C NMR, and HRMS. All the compounds were screened for anti-inflammatory activity based on their inhibitory effects against LPS-induced NO release. Among them, 5-(3,4,5-trimethoxybenzyl)-4-oxo-4,5-dihydro-[1, 2, 4]triazolo[4,3-a]quinoxaline-1-carboxamide (**6p**) showed the highest anti-inflammatory activity and inhibited NO release more potently than the lead compound **D1**. Further studies revealed that compound **6p** reduced the levels of NO, TNF-α, and IL-6, and that its anti-inflammatory activity involves the inhibition of COX-2 and iNOS and downregulation of the mitogen-activated protein kinases (MAPK) signal pathway. Notably, compound **6p** displayed more prominent anti-inflammatory activity than **D1** and the positive control ibuprofen in the *in vivo* acute inflammatory model. Overall, these findings indicate that compound **6p** is a therapeutic candidate for the treatment of inflammation.

## 1. Introduction

Inflammation is a protective host immune response to noxious stimuli, injury, or infection^1^. Long-term chronic inflammation, however, can cause increased vascular permeability, fibrosis, and tissue damage and can lead to the development of metabolic disorders such as rheumatoid arthritis, atherosclerosis, and even cancer^2^^,^^3^. It is well known that the use of chemical drugs to inhibit inflammation is an effective way to alleviate the symptoms of inflammation-related diseases^4^^,^^5^. Nonsteroidal anti-inflammatory drugs (NSAIDs) are still widely used in clinical applications today. However, they usually cause unexpected side effects, such as peptic ulcers, bleeding, mucosal lesions, and nephrotoxicity^6^^,^^7^. It is, therefore, better to find novel anti-inflammatory compounds with fewer adverse reactions.

As a congenital immune cell, macrophages play an important role in the occurrence and development of inflammation. During inflammation, macrophages overexpress bioactive inflammatory mediators such as nitric oxide (NO), interleukin 6 (IL-6), and tumour necrosis factor-alpha (TNF-α)^8^. Furthermore, the accumulation of these inflammatory mediators can cause cell and tissue damage and eventually chronic inflammation^9^. The inhibition of cytokine release from activated macrophages is, therefore, an important mode of action of anti-inflammatory drugs. The mitogen-activated protein kinases (MAPKs) are a family of signal transduction proteins that include extracellular signal-regulated kinase (ERK), c-Jun N-terminal kinases (JNK), and the p38 isoform (p38), which are involved in the classical pathways that modulate immune-mediated inflammatory responses through the corresponding signalling cascades^10^^,^^11^. Activated MAPKs modify the phosphorylation of the threonine/tyrosine motifs, accelerating iNOS, COX-2, and pro-inflammatory cytokine expression (including NO, TNF-α, and IL-6) in the activated macrophages^12–14^. Thus, the pharmacological interference or inhibition of the MAPK signalling pathway can effectively alleviate the occurrence and development of inflammation. Development of anti-inflammatory agents that target the MAPK pathway might be an attractive therapeutic approach.

**Figure 1.**
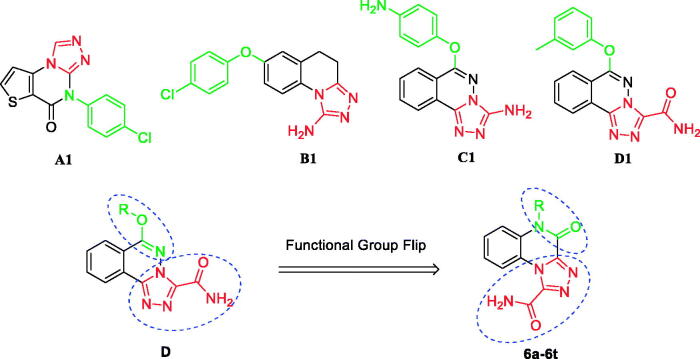
Design of target compounds **6a–6t**.

In previous studies, we presented many different derivative series with significant anti-inflammatory activities in an *in vivo* xylene-induced ear-oedema model^15–18^. A series of 6-substituted-[1, 2, 4]triazolo[3,4-a]phthalazine-3-carboxamide derivatives (**D**) were found to be especially effective anti-inflammatory agents *in vitro* and *in vivo*^18^. Among them, compounds **A1**^15^, **B1**^16^, **C1**^17^, and **D1**^18^ were shown to be the most active derivatives in each series, and their structures are shown in Figure 1. Structure analysis of these derivatives revealed the following: (a) a tricyclic skeleton that contains a triazole ring; and (b) a triazole, 3-amino triazole, or 3-amido triazole that was assumed to be the main pharmacological moiety conferring the anti-inflammatory effect. In addition, functional group transitions and flipping are classic and effective strategies for the rational optimisation of lead compounds^19^.

As part of our continuous efforts to develop better novel anti-inflammatory agents and based on the above observations, a series of 5-alkyl-4-oxo-4,5-dihydro-[1, 2, 4]triazolo[4,3-a]quinoxaline-1-carboxamide derivatives (**6a–6t**) were designed and synthesised using the lead compound **D** and the design principles of skeleton migration and functional group flipping. Moreover, the anti-inflammatory effects of these target compounds were evaluated in RAW264.7 cells following lipopolysaccharide (LPS)-induced NO production. Further, the most potent compound, **6p**, with a 3,4,5-trimethoxybenzyl moiety, was subjected to preliminary studies to determine the mechanisms of action underlying its anti-inflammatory activity by western blotting. Finally, we confirmed the anti-inflammatory effects *in vivo* by using the carrageenan method.

## 2. Materials and methods

### 2.1. Chemistry

All chemicals were purchased from commercial source and used without further purification unless otherwise stated. The reactions were monitored by TLC using Merck Kieselgel 60 F 254 plates and visualised under UV light at 254 nm. Column chromatography was generally performed on silica gel (200 mesh size).^1^H-NMR and ^13^C-NMR spectra were measured on an AV-300 (Bruker BioSpin, Switzerland) and all chemical shifts were given in ppm relative to tetramethylsilane (TMS). High-resolution mass spectra (HRMS) were measured with an Thermo Scientific LTQ Orbitrap XL in ESI mode (Supplementary material).

### 2.2. Procedure for the synthesis of compound 2

A mixture of benzene-1,2-diamine (**1**) (10.0 g, 92 mmol), oxalic acid (12.50 g, 139 mmol), and 30.00 ml 10% HCl in 30 ml H_2_O was stirred at 100 °C for 2 h. The mixture was cooled and then filtered to obtain 1,4-dihydroquinoxaline-2,3-dione (**2**) as a white solid. Yield: 93%, m.p. >300 °C. ^1^H-NMR (DMSO-d_6_, 300 MHz) *δ*: 7.06–7.15 (m, 4H, Ar-H), 11.92 (s, 2H, –CONH–).

### 2.3. Procedure for the synthesis of compound 3

A solution of compound 2 (5.0 g, 46 mmol) in hydrazine hydrate (100 ml) was stirred at 100 °C for 2 h. The mixture was cooled and then filtered to obtain 3-hydrazinylquinoxalin-2(1*H*)-one (**3**) as a yellow solid. Yield: 84%, m.p. >300 °C. ^1^H-NMR (DMSO-d_6_, 300 MHz) *δ*: 4.52 (s, 2H, NH_2_), 7.11–7.37 (m, 4H, Ar-H), 8.37 (s, 1H, –NH–), 12.07 (s, 1H, –CONH–).

### 2.4. Procedure for the synthesis of compound 4

To a suspension of compound **3** (1.3 g, 5 mmol) in diethyl oxalate (15 ml) was stirred at reflux for 3 h. The solvent was removed under reduced pressure to give light yellow oil. The crude residue was purified by silica gel column chromatography (EtOAc) and recrystallized from petroleum ether to give compound **4**, yield: 34%.

### 2.5. General procedure for the synthesis of compound (5a–5t)

A mixture of compound **4** (0.52 g, 2 mmol), K_2_CO_3_ (0.28 g, 2 mmol) and all kinds of halogenated alkanes (2.4 mmol) in DMF (25 ml) was stirred at 50 °C for 3–5 h monitored by TLC and then concentrated under reduced pressure. The residue was added water (50.0 ml) and extracted with dichloromethane. The organic layer was washed with saturated NaCl and dried over anhydrous Na_2_SO_4_. Then it can be purified by chromatography on silica eluting with a gradient of methanol/dichloromethane (1:50) to obtain the compounds **5a–5t** as white solids.

#### 2.5.1. Ethyl 5-benzyl-4-oxo-4,5-dihydro-[1, 2, 4]triazolo[4,3-a]quinoxaline-1-carboxylate (5g)

Yield: 58.9%, m.p. 157–159 °C. ^1^H-NMR (CDCl_3_, 300 MHz) *δ*: 1.57 (t, 3H, *J* = 7.1 Hz, –CH_3_), 4.67 (q, 2H, *J* = 7.1 Hz, –O–CH_2_–C), 5.63 (s, 2H, –CH_2_–), 7.32–7.46 (m, 8H, Ar-H), 8.61 (d, 1H, *J* = 8.3 Hz, Ar-H).

### 2.6 General procedure for the synthesis of compound (6a–6t)

To a stirred solution of compound **5a–5t** (2 mm0l) in MeOH (10 ml) and ammonium hydroxide (10 ml) was added. The reaction mixture was stirred at room temperature for 2 h. The mixture was filtered and then washed with MeOH to give crude product. The resulting solid residue was purified by silica column chromatography (CH_2_Cl_2_/MeOH, 30:1) to give compounds **6a–6t** as pale white solids.

#### 2.6.1. 5-butyl-4-oxo-4,5-dihydro-[1, 2, 4]triazolo[4,3-a]quinoxaline-1-carboxamide (6a)

Yield: 46.4%, m.p. 262–264 °C. IR (KBr) cm^−1^: 3314, 3180, 1693 (C=O), 1665 (C=O). ^1^H-NMR (300 MHz, DMSO-d_6_) *δ*: 0.95 (t, *J* = 7.2 Hz, 3H, –CH_3_), 1.40–1.47 (m, 2H, –CH_2_–), 1.61–1.71 (m, 2H, –CH_2_–), 4.29 (t, *J* = 7.3 Hz, 2H, –N–CH_2_–), 7.41 (t, *J* = 7.8 Hz, 1H, Ar-H), 7.60 (t, *J* = 7.8 Hz, 1H, Ar-H), 7.70 (d, *J* = 8.4 Hz, 1H, Ar-H), 8.47 (d, *J* = 8.4 Hz, 1H, Ar-H), 8.49, 8.88 (s, 2H, –NH_2_). ^13 ^C-NMR (75 MHz, DMSO-d_6_) *δ*: 14.20, 19.98, 29.28, 41.79, 117.10, 119.38, 121.13, 123.74, 129.09, 129.95, 144.31, 146.44, 151.81, 160.53. ESI-HRMS calculated for C_14_H_16_N_5_O_2_^+^ ([M + H]^+^): 286.1299; found: 286.1302.

#### 2.6.2. 5-pentyl-4-oxo-4,5-dihydro-[1, 2, 4]triazolo[4,3-a]quinoxaline-1-carboxamide (6b)

Yield: 56.7%, m.p. 232–234 °C. IR (KBr) cm^−1^: 3354, 3162, 1680 (C=O). ^1^H-NMR (300 MHz, DMSO-d_6_) *δ*: 0.88 (t, *J* = 6.5 Hz, 3H, –CH_3_), 1.38 (s, 4H, –(CH_2_)_2_–), 1.68 (s, 2H, –CH_2_–), 4.28 (t, *J* = 6.2 Hz, 2H, –N–CH_2_–), 7.40 (t, *J* = 7.8 Hz, 1H, Ar-H), 7.60 (t, *J* = 7.8 Hz, 1H, Ar-H), 7.70 (d, *J* = 8.4 Hz, 1H, Ar-H), 8.47 (d, *J* = 8.4 Hz, 1H, Ar-H), 8.49, 8.88 (s, 2H, –NH_2_). ^13 ^C NMR (75 MHz, DMSO-d_6_) *δ*: 14.35, 22.39, 26.88, 28.81, 42.00, 117.09, 119.39, 121.13, 123.75, 129.11, 129.96, 144.32, 146.45, 151.81, 160.53. ESI-HRMS calculated for C_15_H_18_N_5_O_2_^+^ ([M + H]^+^): 300.1455; found: 300.1458.

#### 2.6.3. 5-hexyl-4-oxo-4,5-dihydro-[1, 2, 4]triazolo[4,3-a]quinoxaline-1-carboxamide (6c)

Yield: 36.4%, m.p. 239–241 °C. IR (KBr) cm^−1^: 3360, 3169, 1725 (C=O), 1683 (C=O). ^1^H-NMR (DMSO-d_6_, 300 MHz) *δ*: 0.88 (t, 3H, *J* = 6.3 Hz, –CH_3_) 1.30–1.42 (m, 6H, –(CH_2_)_3_–), 1.61–1.72 (m, 2H, –CH_2_–), 4.27 (t, 2H, *J* = 7.5 Hz, –N–CH_2_–), 7.40 (t, *J* = 7.8 Hz, 1H, Ar-H), 7.59 (t, *J* = 7.8 Hz, 1H, Ar-H), 7.71 (d, *J* = 8.4 Hz, 1H, Ar-H), 8.46 (d, 1H, *J* = 8.4 Hz, Ar-H), 8.50, 8.89 (s, 2H, –NH_2_). ^13 ^C NMR (75 MHz, DMSO-d_6_) *δ*: 14.35, 22.49, 26.33, 27.14, 31.44, 42.04, 117.03, 119.39, 121.11, 123.71, 129.08, 129.92, 144.29, 146.46, 151.77, 160.50. ESI-HRMS calculated for C_16_H_20_N_5_O_2_^+^ ([M + H]^+^): 314.1612; found: 314.1161.

#### 2.6.4. 5-heptyl-4-oxo-4,5-dihydro-[1, 2, 4]triazolo[4,3-a]quinoxaline-1-carboxamide (6d)

Yield: 32.2%, m.p. 231–233 °C. IR (KBr) cm^−1^: 3349, 3156, 1682 (C=O). ^1^H-NMR (300 MHz, DMSO-d_6_) *δ*: 0.85 (s, 3H, *J* = 6.3 Hz, –CH_3_), 1.26–1.38 (m, 8H, –(CH_2_)_4_–), 1.67 (s, 2H, –CH_2_–), 4.27 (s, 2H, –N–CH_2_–), 7.39–7.70 (m, 3H, Ar-H), 8.46 (s, 1H, Ar-H), 8.49, 8.89 (s, 2H, –NH_2_). ^13 ^C-NMR (75 MHz, DMSO-d_6_) *δ*: 14.41, 22.51, 26.64, 27.19, 28.92, 31.68, 42.03, 117.06, 119.39, 121.10, 123.72, 129.08, 129.92, 144.31, 146.45, 151.78, 160.51. ESI-HRMS calculated for C_17_H_22_N_5_O_2_^+^ ([M + H]^+^): 328.1768; found: 328.1765.

#### 2.6.5. 5-octyl-4-oxo-4,5-dihydro-[1, 2, 4]triazolo[4,3-a]quinoxaline-1-carboxamide (6e)

Yield: 30.1%, m.p. 230–232 °C. IR (KBr) cm^−1^: 3351, 3156, 1682 (C=O), 1673(C=O). ^1^H-NMR (300 MHz, DMSO-d_6_) *δ*: 0.85 (s, 3H, *J* = 6.3 Hz, –CH_3_), 1.24 (s, 8H, –(CH_2_)_4_–), 1.40 (s, 2H, –CH_2_–), 1.58–1.74 (m, 2H, –CH_2_–) 4.27 (t, 2H, *J* = 6.6 Hz, –N–CH_2_–), 7.39 (t, 1H, *J* = 7.6 Hz, Ar-H), 7.58 (t, 1H, *J* = 7.6 Hz, Ar-H), 7.69 (d, 1H, *J* = 8.2 Hz, Ar-H), 8.47 (t, 1H, *J* = 8.2 Hz, Ar-H), 8.49, 8.89 (s, 2H, –NH_2_). ^13 ^C NMR (75 MHz, DMSO-d_6_) *δ*: 14.40, 22.54, 26.67, 27.17, 29.09, 29.21, 31.69, 42.03, 117.07, 119.40, 121.12, 123.72, 129.09, 129.93, 144.31, 146.45, 151.78, 160.51. ESI-HRMS calculated for C_18_H_24_N_5_O_2_^+^ ([M + H]^+^): 342.1925; found: 342.1922.

#### 2.6.6. 5-nonyl-4-oxo-4,5-dihydro-[1, 2, 4]triazolo[4,3-a]quinoxaline-1-carboxamide (6f)

Yield: 30.1%, m.p. 236–238 °C. IR (KBr) cm^−1^: 3353, 3155, 1682 (C=O), 1671(C=O). ^1^H-NMR (300 MHz, DMSO-d_6_) *δ*: 0.84 (m, 3H, *J* = 6.3 Hz, –CH_3_), 1.24–1.40 (m, 12H, –(CH_2_)_6_–), 1.58–1.74 (m, 2H, –CH_2_–), 4.26 (t, 2H, *J* = 6.8 Hz, –N–CH_2_–), 7.39 (t, 1H, *J* = 7.6 Hz, Ar-H), 7.58 (t, 1H, *J* = 7.6 Hz, Ar-H), 7.69 (d, 1H, *J* = 8.2 Hz, Ar-H), 8.47 (t, 1H, *J* = 8.2 Hz, Ar-H), 8.49, 8.89 (s, 2H, –NH_2_). ^13 ^C NMR (75 MHz, DMSO-d_6_) *δ*: 14.41, 22.55, 26.66, 27.17, 29.11, 29.26, 29.36, 31.74, 42.02, 117.07, 119.40, 121.12, 123.72, 129.08, 129.93, 144.31, 146.45, 151.78, 160.51. ESI-HRMS calculated for C_19_H_26_N_5_O_2_^+^ ([M + H]^+^): 356.2081; found: 356.2078.

#### 2.6.7. 5-benzyl-4-oxo-4,5-dihydro-[1, 2, 4]triazolo[4,3-a]quinoxaline-1-carboxamide (6g)

Yield: 35.8%, m.p. 268–270 °C. IR (KBr) cm^−1^: 3345, 3157, 1682 (C=O). ^1^H-NMR (300 MHz, DMSO-d_6_) *δ*: 5.57 (s, 2H, –N–CH_2_–), 7.36–7.45 (m, 8H, Ar-H), 8.52 (s, 2H, Ar-H, –NH), 8.92 (s, 1H, –NH). ^13 ^C NMR (75 MHz, DMSO-d_6_) *δ*: 45.42, 117.56, 119.35, 121.38, 123.96, 127.06 (2 C), 127.77, 128.85, 129.12 (2 C), 130.10, 136.07, 144.59, 146.60, 152.54, 160.53. ESI-HRMS calculated for C_17_H_14_N_5_O_2_^+^ ([M + H]^+^): 320.1142; found: 320.1140.

#### 2.6.8. 5-(2-fluorobenzyl)-4-oxo-4,5-dihydro-[1, 2, 4]triazolo[4,3-a]quinoxaline-1-carboxamide (6h)

Yield: 28.2%, m.p. 273–275 °C. IR (KBr) cm^−1^: 3340, 3165, 1685 (C=O). ^1^H-NMR (300 MHz, DMSO-d_6_) *δ*: 5.56 (s, 2H, –N–CH_2_–), 7.08–7.50 (m, 7H, Ar-H), 8.54 (m, 2H, Ar-H, –NH), 8.95 (s, 1H, –NH). ^13 ^C NMR (75 MHz, DMSO-d_6_) *δ*: 44.05, 115.90 (d, *J* = 20.8 Hz), 117.11, 119.42, 121.45, 122.81 (d, *J* = 14.0 Hz), 124.09, 125.10 (d, *J* = 2.5 Hz), 128.60 (d, *J* = 3.5 Hz), 129.01, 129.88 (d, *J* = 7.5 Hz), 130.08, 144.58, 146.60, 152.50, 160.33 (d, *J* = 242.9 Hz), 160.50. ESI-HRMS calculated for C_17_H_13_FN_5_O_2_^+^ ([M + H]^+^): 338.1048; found: 338.1044.

#### 2.6.9. 5–(3-fluorobenzyl)-4-oxo-4,5-dihydro-[1, 2, 4]triazolo[4,3-a]quinoxaline-1- carboxamide (6i)

Yield: 32.4%, m.p. 276–278 °C. IR (KBr) cm^−1^: 3342, 3155, 1685 (C=O). ^1^H-NMR (300 MHz, DMSO-d_6_) *δ*: 5.57 (s, 2H, –N–CH_2_–), 7.10 (t, *J* = 8.0 Hz, 1H, Ar-H), 7.25 (t, *J* = 7.1 Hz, 2H, Ar-H), 7.35–7.49 (m, 4H, Ar-H), 8.51 (s, 1H, Ar-H), 8.54, 8.93 (s, 2H, –NH_2_). ^13 ^C-NMR (75 MHz, DMSO-d_6_) *δ*: 45.09, 114.17 (d, *J* = 38.6 Hz), 114.46 (d, *J* = 37.3 Hz), 117.37, 119.38, 121.53, 123.15, 123.97, 128.84, 130.10, 131.1 (d, *J* = 8.2 Hz), 139.12 (d, *J* = 7.2 Hz), 144.76, 146.55, 152.65, 160.54, 162.93 (d, *J* = 242.1 Hz). ESI-HRMS calculated for C_17_H_13_FN_5_O_2_^+^ ([M + H]^+^): 338.1048; found: 338.1047.

#### 2.6.10. 5-(4-fluorobenzyl)-4-oxo-4,5-dihydro-[1, 2, 4]triazolo[4,3-a]quinoxaline-1-carboxamide (6j)

Yield: 25.7%, m.p. 274–276 °C. IR (KBr) cm^−1^: 3342, 1685 (C=O). ^1^H-NMR (300 MHz, DMSO-d_6_) *δ*: 5.54 (s, 2H, –N–CH_2_–), 7.16 (t, *J* = 8.8 Hz, 2H, Ar-H), 7.35-8.46 (m, 5H, Ar-H), 8.50 (s, 1H, Ar-H), 8.52, 8.91 (s, 2H, –NH_2_). ^13 ^C-NMR (75 MHz, DMSO-d_6_) *δ*: 44.79, 115.88 (d, *J* = 21.4 Hz, 2 C), 117.46, 119.37, 121.44, 123.97, 128.86, 129.27 (d, *J* = 8.1 Hz, 2 C), 130.04, 132.24 (d, *J* = 2.7 Hz), 144.64, 146.57, 152.57, 160.52. 161.87 (d, *J* = 241.7 Hz), ESI-HRMS calculated for C_17_H_13_FN_5_O_2_^+^ ([M + H]^+^): 338.1048; found: 338.1045.

#### 2.6.11. 5-(2-chlorobenzyl)-4-oxo-4,5-dihydro-[1, 2, 4]triazolo[4,3-a]quinoxaline-1-carboxamide (6k)

Yield: 31.6%, m.p. 278–280 °C. IR (KBr) cm^−1^: 3334, 3164, 1685 (C=O). ^1^H-NMR (300 MHz, DMSO-d_6_) *δ*: 5.51 (s, 2H, –N–CH_2_–), 7.14–7.23 (m, 3H, Ar-H), 7.32–7.51 (m, 3H, Ar-H), 7.59 (d, *J* = 7.9, 1H, Ar-H), 8.53 (s, 1H, Ar-H), 8.56, 8.95 (s, 2H, –NH_2_). ^13 ^C-NMR (75 MHz, DMSO-d_6_) *δ*: 44.24, 117.11, 119.45, 121.55, 124.11, 127.81, 127.94, 129.07, 129.58, 130.00, 130.16, 132.09, 132.81, 144.70, 146.60, 152.53, 160.51. ESI-HRMS calculated for C_17_H_13_ClN_5_O_2_^+^ ([M + H]^+^): 354.0752; found: 354.0749.

#### 2.6.12. 5-(3-chlorobenzyl)-4-oxo-4,5-dihydro-[1, 2, 4]triazolo[4,3-a]quinoxaline-1-carboxamide (6 l)

Yield: 25.8%, m.p. 269–271 °C. IR (KBr) cm^−1^: 3338, 3158, 1685 (C=O). ^1^H-NMR (300 MHz, DMSO-d_6_) *δ*: 5.56 (s, 2H, –N–CH_2_–), 7.36–7.51 (m, 7H, Ar-H), 8.52 (s, 1H, Ar-H), 8.54, 8.92 (s, 2H, –NH_2_). ^13 ^C-NMR (75 MHz, DMSO-d_6_) *δ*: 45.07, 117.36, 119.38, 121.55, 123.98, 125.82, 126.94, 127.80, 128.87, 130.14, 130.93, 133.91, 138.77, 146.54, 152.69, 160.53. ESI-HRMS calculated for C_17_H_13_ClN_5_O_2_^+^ ([M + H]^+^): 354.0752; found: 354.0748.

#### 2.6.13. 5-(4-chlorobenzyl)-4-oxo-4,5-dihydro-[1, 2, 4]triazolo[4,3-a]quinoxaline-1-carboxamide (6m)

Yield: 20.3%, m.p. 282–284 °C. IR (KBr) cm^−1^: 3334, 3158, 1685 (C=O). ^1^H-NMR (300 MHz, DMSO-d_6_) *δ*: 5.55 (s, 2H, –N–CH_2_–), 7.40 (s, 7H, Ar-H), 8.52 (s, 2H, Ar-H, –NH), 8.93 (s, 1H, –NH). ^13 ^C-NMR (75 MHz, DMSO-d_6_) *δ*: 44.88, 117.43, 119.38, 121.44, 124.01, 128.88, 129.03 (2 C), 129.10 (2 C), 130.02, 132.38, 135.17, 144.64, 146.57, 152.57, 160.52. ESI-HRMS calculated for C_17_H_13_ClN_5_O_2_^+^ ([M + H]^+^): 354.0752; found: 354.0750.

#### 2.6.14. 5-(3-methoxybenzyl)-4-oxo-4,5-dihydro-[1, 2, 4]triazolo[4,3-a]quinoxaline-1-carboxamide (6n)

Yield: 38.3%, m.p. 266–268 °C. IR (KBr) cm^−1^: 3314, 3191, 1691 (C=O), 1668(C=O). ^1^H-NMR (300 MHz, DMSO-d_6_) *δ*: 3.72 (s, 3H, –O–CH_3_), 5.52 (s, 2H, –N–CH_2_–), 6.82–6.97 (m, 3H, Ar-H), 7.23 (t, *J* = 8.8 Hz, 1H, Ar-H), 7.35-7.47 (m, 3H, Ar-H), 8.50 (s, 1H, Ar-H), 8.52, 8.91 (s, 2H, –NH_2_). ^13 ^C-NMR (75 MHz, DMSO-d_6_) *δ*: 45.43, 55.51, 112.87, 113.16, 117.56, 119.03, 119.30, 121.39, 123.94, 128.84, 130.18, 130.26, 137.71, 144.62, 146.58, 152.55, 160.02, 160.53. ESI-HRMS calculated for C_18_H_16_N_5_O_3_ ([M + H]^+^): 350.1248; found: 350.1249.

#### 2.6.15. 5-(4-methoxybenzyl)-4-oxo-4,5-dihydro-[1, 2, 4]triazolo[4,3-a]quinoxaline-1- carboamide (6o)

Yield: 42.5%, m.p. 264–266 °C. IR (KBr) cm^−1^: 3340, 1682 (C=O). ^1^H-NMR (300 MHz, DMSO-d_6_) *δ*: 3.71 (s, 3H, –O–CH_3_), 5.49 (s, 2H, –N–CH_2_–), 6.89 (s, 2H, Ar-H), 7.33–7.48 (m, 5H, Ar-H), 8.51 (s, 2H, Ar-H, –NH), 8.91 (s, 1H, –NH). ^13 ^C NMR (75 MHz, DMSO-d_6_) *δ*: 44.85, 55.51, 114.51 (2 C), 117.60, 119.33, 121.33, 123.91, 127.86, 128.55 (2 C), 128.82, 130.04, 144.55, 146.59, 152.49, 158.96, 160.53. ESI-HRMS calculated for C_18_H_16_N_5_O_3_ ([M + H]^+^): 350.1248; found: 350.1251.

#### 2.6.16. 5-(3,4,5-trimethoxybenzyl)-4-oxo-4,5-dihydro-[1, 2, 4]triazolo[4,3-a]quinoxali- ne-1-carboxamide (6p)

Yield: 50.6%, m.p. 286–288 °C. IR (KBr) cm^−1^: 3317, 1688 (C=O), 1665(C=O). ^1^H-NMR (300 MHz, DMSO-d_6_) *δ*: 3.62 (s, 3H, –O–CH_3_), 3.71 (s, 6H, –O–CH_3_), 5.46 (s, 2H, –N–CH_2_–), 6.73 (s, 2H, Ar-H), 7.39–8.50 (m, 3H, Ar-H), 8.52 (s, 1H, Ar-H), 8.54, 8.92 (s, 2H, –NH_2_). ^13 ^C NMR (75 MHz, DMSO-d_6_) *δ*: 45.96, 56.49 (2 C), 60.44, 104.79 (2 C), 117.57, 119.28, 121.40, 123.90, 128.84, 130.39, 131.91, 137.16, 144.77, 146.57, 152.69, 153.61 (2 C), 160.55. ESI-HRMS calculated for C_20_H_20_N_5_O_5_ ([M + H]^+^): 410.1459; found: 410.1461.

#### 2.6.17. 5-(2,4-dichlorobenzyl)-4-oxo-4,5-dihydro-[1, 2, 4]triazolo[4,3-a]quinoxaline- 1-carboxamide (6q)

Yield: 26.3%, m.p. 266–268 °C. IR (KBr) cm^−1^: 3342, 3160, 1682 (C=O). ^1^H-NMR (300 MHz, DMSO-d_6_) *δ*: 5.47 (s, 2H, –N–CH_2_–), 7.21–7.27 (m, 3H, Ar-H), 7.40–7.48 (m, 2H, Ar-H), 7.77 (s, 1H, Ar-H), 8.53 (s, 1H, Ar-H), 8.56, 8.96 (s, 2H, –NH_2_). ^13 ^C-NMR (75 MHz, DMSO-d_6_) *δ*: 44.01, 117.06, 119.46, 121.59, 124.15, 127.98, 129.08, 129.29, 129.50, 130.09, 132.17, 133.13, 133.26, 144.73, 146.59, 152.55, 160.49. ESI-HRMS calculated for C_17_H_12_Cl_2_N_5_O_2_ ([M + H]^+^): 388.0363; found: 388.0367.

#### 2.6.18. 5-(3-(Trifluoromethyl)benzyl)-4-oxo-4,5-dihydro-[1, 2, 4]triazolo[4,3-a]quinox- aline-1-carboxamide (6r)

Yield: 36.7%, m.p. 262–264 °C. IR (KBr) cm^−1^: 3331, 3165, 1685 (C=O). ^1^H-NMR (300 MHz, DMSO-d_6_) *δ*: 5.65 (s, 2H, –N–CH_2_–), 7.35–7.50 (m, 3H, Ar-H), 7.56 (d, *J* = 7.6 Hz, 1H, Ar-H), 7.66 (t, *J* = 7.0 Hz, 2H, Ar-H), 7.85 (s, 1H, Ar-H), 8.52 (s, 1H, Ar-H), 8.55, 8.93 (s, 2H, –NH_2_). ^13 ^C-NMR (75 MHz, DMSO-d_6_) *δ*: 45.28, 117.29, 119.42, 121.55, 122.82, 124.01, 124.14, 124.57 (d, *J* = 4.4 Hz), 128.03 (d, *J* = 240.5 Hz), 128.89, 129.84 (d, *J* = 31.7 Hz), 130.14 (d, *J* = 6.1 Hz), 131.14, 137.76, 144.81, 146.56, 152.76, 160.54. ESI-HRMS calculated for C_18_H_13_F_3_N_5_O_2_ ([M + H]^+^): 388.1016; found: 388.1018.

#### 2.6.19. 5-(4-nitrobenzyl)-4-oxo-4,5-dihydro-[1, 2, 4]triazolo[4,3-a]quinoxaline-1-carb- oxamide (6 s)

Yield: 25.1%, m.p. 273–275 °C. IR (KBr) cm^−1^: 3465, 3311, 1674 (C=O). ^1^H-NMR (300 MHz, DMSO-d_6_) *δ*: 5.70 (s, 2H, –N–CH_2_–), 7.36–7.48 (m, 3H, Ar-H), 7.68 (d, *J* = 8.4 Hz, 2H, Ar-H), 8.18 (d, *J* = 8.5 Hz, 2H, Ar-H), 8.52 (s, 1H, Ar-H), 8.55, 8.96 (s, 2H, –NH_2_). ^13 ^C-NMR (75 MHz, DMSO-d_6_) *δ*: 45.27, 117.29, 119.47, 121.54, 124.15, 128.37 (3 C), 128.94 (2 C), 130.02, 144.18, 144.71, 146.60, 147.28, 152.65, 160.50. ESI-HRMS calculated for C_17_H_13_N_6_O_4_ ([M + H]^+^): 365.0993; found: 365.0996.

#### 2.6.20. 5-(4-cyanobenzyl)-4-oxo-4,5-dihydro-[1, 2, 4]triazolo[4,3-a]quinoxaline-1-carboxamide (6t)

Yield: 23.6%, m.p. 298–300 °C. IR (KBr) cm^−1^: 3440, 3194, 1680 (C=O). ^1^H-NMR (300 MHz, DMSO-d_6_) *δ*: 5.65 (s, 2H, –N–CH_2_–), 7.38–7.46 (m, 3H, Ar-H), 7.60 (d, *J* = 7. 3 Hz, 2H, Ar-H), 7.82 (d, *J* = 7.3 Hz, 2H, Ar-H), 8.52 (s, 1H, Ar-H), 8.54, 8.94 (s, 2H, –NH_2_). ^13 ^C-NMR (75 MHz, DMSO-d_6_) *δ*: 45.38, 110.60, 117.28, 119.19, 119.45, 121.55, 124.07, 128.10 (2 C), 128.91, 130.05, 132.99 (2 C), 142.05, 144.74, 146.58, 152.66, 160.51. ESI-HRMS calculated for C_18_H_13_N_6_O_2_ ([M + H]^+^): 345.1095; found: 345.1097.

### 2.7. Cell culture and sample treatment

The RAW 264.7 mouse macrophage cell line was obtained from the China Cell Line Bank (Beijing, China). Cells were cultured in complete medium (DMEM supplemented with 10% heat-inactivated FBS, 3 mM glutamine and antibiotics (100 U/mL penicillin and 100 U/mL streptomycin)) at 37 °C in a humidified incubator containing 5% CO_2_ and 95% air. Cells were treated with various concentrations of synthesised compounds for 30 min followed by stimulation with LPS (1 µg/L).

### 2.8. MTT assay for cell viability

Cell viability studies induced by synthesised compounds were evaluated by 3-(4,5-dimethylthiazol-2-yl)-2,5-diphenyltetrazolium bromide (MTT) assay. RAW264.7 macrophages were seeded in 96-well plates at a density of 5 × 10^5^ cells/mL in complete medium and incubated for 24 h (100 µL/well). Then the cells were treated with different concentrations of synthesised compounds for 24 h. 150 µL MTT (5 g/L in PBS) was added to each well and the cells were further incubated for 4 h. The supernatant was removed and the cells were lysed with 150 µL/well DMSO. The optical density was measured at 570 nm on a microplate reader (Thermo Scientific, MA, USA).

### 2.9. Assay for NO production

Nitrite, a stable product of nitric oxide, was used to assess NO production. To study the effects of the isolated compounds on NO production, RAW264.7 cells were plated at 1 × 10^5^ cells/well in a 96-well microplate and incubated overnight. The cells were pre-treated with various concentrations of the test compounds (3, 7.5, 10, 15, 30 or 100 µM) and further co-cultured with 1 µg/mL LPS for 24 h. Un-pre-treated and un-stimulated RAW 264.7 cells were conducted as the blank control group. At different time points, the supernatant (50 µL) was assayed with NO assay kit. The culture supernatant (50 µL) was mixed with an equal volume of the Griess reagent (1% sulphanilamide, 0.1% N-[1-naphthy] ethylenediamine dihydrochloride) and NO production were determined at 540 nm with an ELISAplate reader.

### 2.10. Cytokine production assay

RAW264.7 cells were seeded at 3 × 10^5^ cells/well in a 48-well microplate and incubated overnight.The cells were pre-treated with the different concentrations of the test compunds (3, 7.5, 10 or 30 µM) for 30 min before the stimulation with LPS (1 µg/mL). According to the protocol of the manufacturer, supernatants were harvested at the 24th hour for the assay of TNF-α or IL-6 with ELISA kits (BD Biosciences, San Diego, CA, USA). Briefly, 100 µL of biotinylated antibody reagent and the culture supernatants were added to anti-mouse TNF-α or IL-6 precoated 96-well plates, and the plates were incubated for 2 h at RT. The plate was washed with a washing buffer and subsequently incubated with 100 µL of the streptavidin-HRP solution for 1 h at RT. The plate was washed, and incubated with 100 µL of TMB substrate solution for 30 min at RT in the dark. The reaction was stopped by adding 50 µL of stop solution, and then the absorbance was measured at 450 nm by a plate reader. A standard curve was produced for each assay plate using serially diluted recombinant IL-6.

### 2.11. Western blot analysis

RAW 264.7 cells (5 × 10^5^ cells/well) plated onto 6-well plates were incubated for 24 h and treated with 7.5, 15.0 or 30.0 µM of compound **6p** for 30 min and then stimulated with 1 µg/L of LPS for 24 h. The cells were collected and washed three times with ice-cold PBS. The cells were treated with a cell lysis buffer [50 mM Tris (pH 7.6), 150 mM NaCl, 5 mM EDTA (pH 8.0), 0.6% NP-40, 1 mM Na_3_VO_4_, 20 mM β-glycerophosphate, 1 mM phenylmethylsulfonyl fluoride, 2 mM p-nitrophenyl phosphate, and 1:25 Complete Mini Protease Inhibitor cocktail (Boehringer, Mannheim,Germany)] and kept on ice for 30 min. The cell lysates were centrifuged (12,000 g at 4 °C) for 5 min to obtain a cytosolic fraction. The protein concentration was determined by BCA protein assay kit (Beyotime, Haimen, China). Aliquots of the lysates were separated on 10% sodium dodecyl sulphate (SDS)-polyacrylamide gel electrophoresis (PAGE) and then electro blotted onto a polyvinylidene difluoride (PVDF) membrane. The blots were blocked with 5% (w/v) non-fat dry milk for 2 h at 37 °C, followed by incubation with specific primary antibody at 4 °C overnight. Blots were washed with Tween 20/Tris-buffered saline [TTBS, 20 mM Tris–HCl buffer, pH 7.6, containing 137 mM NaCl and 0.05% (vol/vol) Tween 20] and incubated with a peroxidase-conjugated secondary antibody for 1 h. Blots were again washed with TTBS and the immune active proteins were detected using ECL plus (Thermo, USA). All Western blot analyses were carried out at least three times. Results are expressed as the relative ratio of the specific band compared with the internal reference.

### 2.12. In vivo anti-inflammatory activities determination

The animal procedures were in strict accordance with the National Institutes of Healthy Guidelines for the Care and Use of Laboratory Animals (NIH Publication No. 85-23, revised 1996) and were approved by the Institutional Animal Care and Utilisation Committee of Yanbian University. Male albino rats weighing 280–300 gwere obtained from Animal Department of Yanbian University . They were kept in the animal house under standard condition of light and temperature with free access to food and water. The animals were randomly divided into five groups (control, **D1**, **6p**, ibuprofen and celecoxib group) The groups with **D1**, **6p**, ibuprofen and celecoxib were administered oral administration (p.o.) 25 mg/kg in a vehicle of 0.5% methylcellulose, respectively. The negative control group was treated with the same vehicle (0.5% methylcellulose). A solution of carrageenan (0.1 ml, 1.0% w/v in 0.9% of normal saline) was injected into the subplanter region of the left hind paw under light ether anaesthesia 1 h after oral administration (p.o.) of the test compound (at a dose level of 25 mg/kg body weight). The paw volume was measured at hourly interval for 5 h (0, 1, 3 and 5 h) and the percent inhibition of oedema was calculated using formula:

%Inhibition=(Vt−Vo)control−(Vt−Vo)tested compound /(Vt−Vo)control×100

where,

Vo = volume of oedema at zero time interval.
Vt = volume of oedema at specific time interval.

### 2.13. Ulcerogenic effect

The lead compound **D1** and the newly synthesised compound **6p** were subjected to *in vivo* testing to measure their ulcerogenic effect in comparison to celecoxib and ibuprofen. Male albino rats (220–250 g) divided into 5 groups (control, **D1**, **6p**, ibuprofen and celecoxib group) of five rats each. The groups with **D1**, **6p**, ibuprofen, and celecoxib were administered oral administration (p.o.) 25 mg/kg in a vehicle of 0.5% methylcellulose, respectively. The negative control group was treated with the same vehicle (0.5% methylcellulose). All groups were orally administered once a day for three consecutive days. Animals were sacrificed by diethyl ether 6 h after the last dose and the stomach was removed. An opening at the greater curvature was made and the stomach was cleaned by washing with cold saline and inspected with a three-time magnifying lens for any evidence of hyperaemia, haemorrhage, definite haemorrhagic erosion, or ulcer. An arbitrary scale was used to calculate the ulcer index which indicates the severity of the stomach lesions The % ulceration for each group was calculated as follows: % Ulceration = Number of animals bearing ulcer in a group/Total number of animals in the same group ×100.

## 3. Results and discussion

### 3.1. Chemistry

**Scheme 1.**
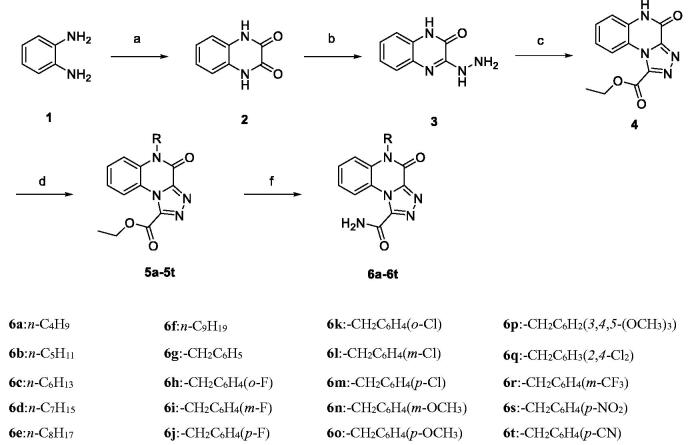
Reagents and conditions: (a) oxalic acid, HCl/H_2_O, 100 °C, 2 h; (b) Hydrazine hydrate, 100 °C, 2 h; (c) Diethyl oxalate, reflux, 3 h; (d) RX, K_2_CO_3_, DMF, 60 °C, 3 h; (e) NH_3_·H_2_O, methanol, r.t., 2 h.

The steps involved in the preparation of the target compounds **6a–6t** are outlined in Scheme 1. The starting material, *o*-phenylenediamine (**1**), was reacted with oxalic acid in 10% hydrochloric acid to obtain quinoxaline-2,3(1*H*,4*H*)-dione (**2**) (a white needle solid). Unlike in the previous synthesis method^20^, diethyl oxalate was replaced with hydrochloric acid solution in oxalic acid, and the white needle-like solid was directly precipitated after the reaction, which not only shortened the reaction time but also made the post-treatment easier. Compound **2** was then reacted with hydrazine hydrate to obtain compound **3** (3-hydrazinylquinoxalin-2(1*H*)-one) with only one carbonyl group substituted^21^, and compound **4** was prepared by cyclizing compound **3** with diethyl oxalate. Finally, compound **4** was reacted with appropriate brominated alkanes and substituted with chlorobenzyl in the presence of K_2_CO_3_ to yield compounds **5a–5t,** which were reacted further with NH_3_·H_2_O in methanol to obtain the target compounds **6a–6t**^18^. The synthesised compounds were analysed by ^1^H-NMR, ^13 ^C-NMR, and HRMS.

### 3.2. Cytotoxicity of the target compounds 6a–*6t*

To investigate whether the anti-inflammatory activities of the target compounds **6a–6t** were related to cell viability, their cytotoxic effects were evaluated by MTT assay in RAW264.7 cells^22^. As shown in Table 1, except for compounds **6i** and **6j**, the other compounds at concentrations of 10 or 30 µM showed no obvious cytotoxic effects in RAW264.7 cells, and the relative cell viabilities of the treated cells were more than 80%. Thus, 10 µM concentration was chosen for subsequent experiments.

**Table 1.** Effect of compounds **6a–6t** on the viability of RAW264.7 cells.

Compound	R	Cell viability (%)	
		30 μM	10 μM
blank	–	100	100
LPS	–	98.60 ± 2.12^a^	NT^b^
**D1**	–	96.30 ± 1.72	NT
**6a**	*n*-C_4_H_9_	92.33 ± 1.53	NT
**6b**	*n*-C_5_H_11_	97.95 ± 1.1	NT
**6c**	*n*-C_6_H_13_	99.11 ± 0.69	NT
**6d**	*n*-C_7_H_15_	81.00 ± 1.10	NT
**6e**	*n*-C_8_H_17_	99.51 ± 2.30	NT
**6f**	*n*-C_9_H_19_	60.00 ± 3.40	99.15 ± 1.65
**6g**	–CH_2_C_6_H_5_	85.02 ± 1.00	NT
**6h**	–CH_2_C_6_H_4_(*o*-F)	66.67 ± 1.33	98.49 ± 1.76
**6i**	–CH_2_C_6_H_4_(*m*-F)	59.33 ± 2.52	99.46 ± 4.17
**6j**	–CH_2_C_6_H_4_(*p*-F)	48.00 ± 3.00	73.45 ± 2.27
**6k**	–CH_2_C_6_H_4_(*o*-Cl)	46.33 ± 2.89	74.56 ± 1.38
**6l**	–CH_2_C_6_H_4_(*m*-Cl)	51.67 ± 2.12	87.92 ± 5.18
**6m**	–CH_2_C_6_H_4_(*p*-Cl)	44.67 ± 3.06	82.18 ± 1.74
**6n**	–CH_2_C_6_H_4_(*m*-OCH_3_)	93.33 ± 0.58	NT
**6o**	–CH_2_C_6_H_4_(*p*-OCH_3_)	80.12 ± 1.00	NT
**6p**	–CH_2_C_6_H_2_(*3*,*4*,*5*-(OCH_3_)_3_)	83.67 ± 1.53	NT
**6q**	–CH_2_C_6_H_3_(*2*,*4*-Cl_2_)	56.67 ± 1.34	84.69 ± 6.49
**6r**	–CH_2_C_6_H_4_(*m*-CF_3_)	99.30 ± 0.60	NT
**6s**	–CH_2_C_6_H_4_(*p*-NO_2_)	80.10 ± 1.34	NT
**6t**	–CH_2_C_6_H_4_(*p*-CN)	90.33 ± 1.21	NT

^a^ LPS (1 μg/mL).

^b^ No tested.

### 3.3. Inhibition of NO production in (LPS)-stimulated RAW264.7 cells and SAR studies

**Figure 2.**
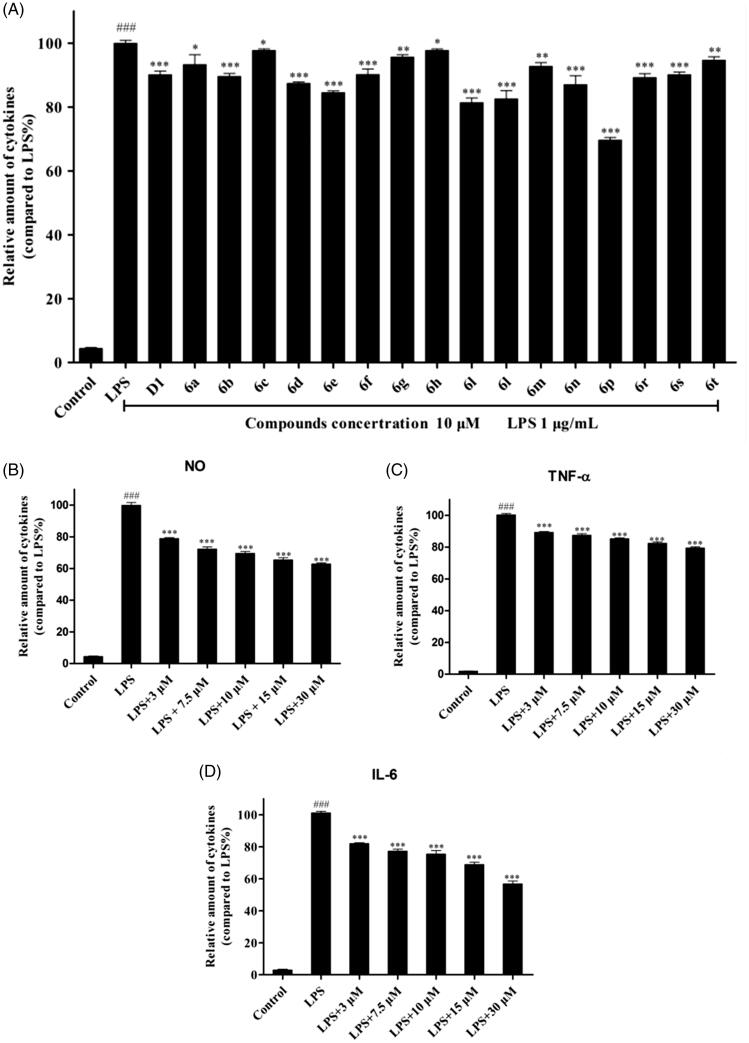
RAW264.7 cells were pre-cultured for 24 h, the cells were then treated with the indicated concentrations of compounds for 30 min, and then exposed to 1 μg/mL LPS for 24 h. The levels of NO in the culture medium were measured with NO assay kit. (A) Cells were treated with 10 μM compounds. Cells were pre-treated with different concentrations of compound **6p**. NO, TNF-α and IL-6 levels in the medium were determined with an ELISA kit. (B, C and D). ### *p* < .01, vs. Control. *p < .05, **p < .01, ***p < .001 vs. LPS alone.

High levels of NO are produced in response to LPS (1 µg/mL) in the activated RAW264.7 macrophages^23^. Therefore, NO inhibitors have been identified as good options for the treatment of inflammatory diseases^24^. The anti-inflammatory activities of the target compounds **6a–6t** were tested based on their ability to inhibit LPS-induced NO production in RAW264.7 macrophages. As shown in Figure 1(A), LPS treatment caused a significant increase in NO release compared to that in untreated controls, which was inhibited after treatment with the synthetic derivatives in RAW264.7 cells. Among them, compounds **6b**, **6d**, **6e**, **6f**, **6i**, **6j**, **6n**, **6o**, **6p**, **6r,** and **6s** exhibited similar or better NO inhibitory activity than the lead compound **D1**. Compound **6p** (10 µM) exhibited the most potent inhibitory activity (38.82%) and was more potent than **D1** (a 10.31% inhibition). When the concentration of compound **6p** was reduced to 3 µM, it was still able to significantly inhibit NO production (*p* < .001) (Figure 2(A)).

The following structure-activity relationships (SAR) of the synthetic compounds were determined: the length of the alkyl chain appeared to have a direct impact on the anti-inflammatory activity of the 5-alkyl derivatives **6a–6f**. As the alkyl chain length of the derivatives **6a–6e** increased, their anti-inflammatory activities increased, with compound **6e** (n-octyl substitution) being the most active. When the length of the linear alkyl group reached 9 carbon atoms (compound **6f**), the anti-inflammatory activity began to decrease. We hypothesised that the n-octyl-substituted compound, **6e,** has the most favourable exponential partition coefficient and easily passes through the cell membrane. For the benzyloxy-substituted derivatives **6g–6t**, the type and position of the substituent on the benzene ring had a significant effect on anti-inflammatory activity. The activity order was *3,4,5*-OCH_3_>*m*-OCH_3_>*p*-OCH_3_>*m*-F>*p*-F>*m*-CF_3_>*p*-NO_2_>*p*-CN, indicating that the electron-donating group exerts a greater effect than the electron-withdrawing group in increasing the anti-inflammatory activities of these derivatives.

### 3.4. *Compound 6p inhibited IL-6 and TNF-*α *production in RAW264.7 cells*

Based on the initial screening results of these synthetic derivatives, compound **6p** was chosen for further assessment of its anti-inflammatory activity in LPS-induced macrophages. It is well known that the pro-inflammatory mediators IL-6 and TNF-α play important roles in the development of inflammation-related diseases^25^. To measure the effects of compound **6p** on LPS-induced IL-6 and TNF-α production, RAW264.7 cells were cultured with LPS (1 µg/mL) in the presence of compound **6p** for 24 h, and the levels of IL-6 and TNF-α in the supernatant were determined by ELISA. As shown in Figure 2(B,C), LPS stimulation significantly elevated the production of TNF-α and IL-6. After treatment with compound **6p,** the LPS-induced increase in IL-6 and TNF-α levels was inhibited in a concentration-dependent manner. Even at low concentrations (3 µM), **6p** significantly inhibited the production of these two inflammatory factors (*p* < .001).

### 3.5. Compound 6p inhibited the expression of iNOS and COX-2

**Figure 3.**
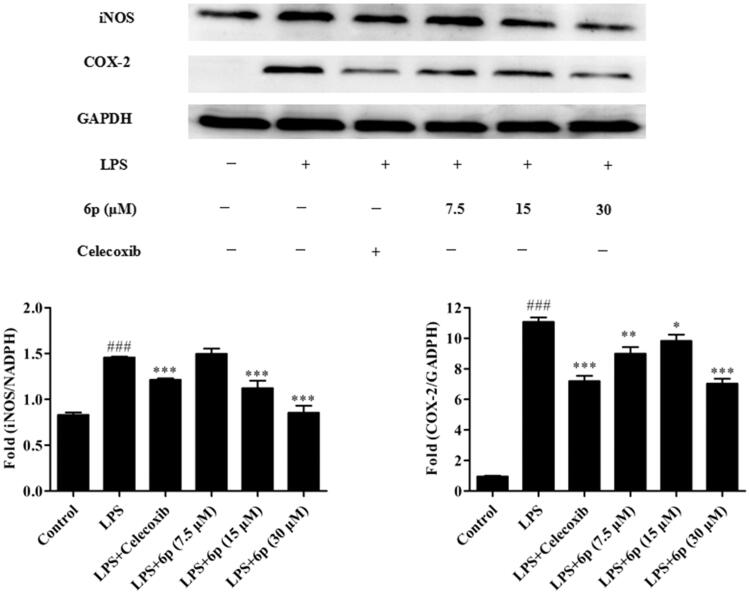
Compound **6p** inhibited LPS-induced inflammatory response in RAW 264.7 cells. Cells were pre-treated with different concentrations of **6p** (7.5–30 µM) and Celecoxib (7.5 µM) for 24 h. iNOS, COX-2 and GAPDH proteins expression were detected by Western blot analysis. Total cellular proteins were prepared and analysed by Western blotting. Results are the mean ± SD, *n* = 3. ### *p* < .001, vs. Control. *p < .05, **p < .01, ***p < .001 vs. LPS alone.

The pro-inflammatory mediator NO plays an important role in inflammation-related diseases and its production is closely related to the modulation of iNOS and COX-2 expression^2^. Our results show that compound **6p** can significantly inhibit the production of NO. To establish whether this inhibitory effect was associated with the reduced expression of iNOS and COX-2 in LPS-induced RAW 264.7 cells, the levels of these proteins were measured by western blotting after exposure to LPS for 24 h in the presence or absence of compound **6p** (7.5, 15, and 30 µM). Celecoxib (7.5 µM) was used as a positive control. As shown in Figure 3, LPS (1 µg/mL) stimulation markedly increased COX-2 and iNOS protein expression and compound **6p** inhibited this response (*p* < .001) by 53.44% (COX-2) at 15 µM and 39.89% (iNOS) at 30 µM, respectively. Moreover, compound **6p** inhibited iNOS protein expression in LPS-induced cells in a dose-dependent manner. These results suggest that **6p** exerts its anti-inflammatory activity through inhibiting the expression of iNOS, and COX-2 by LPS in macrophages.

### 3.6. Compound 6p inhibited MAPK signalling activation

**Figure 4.**
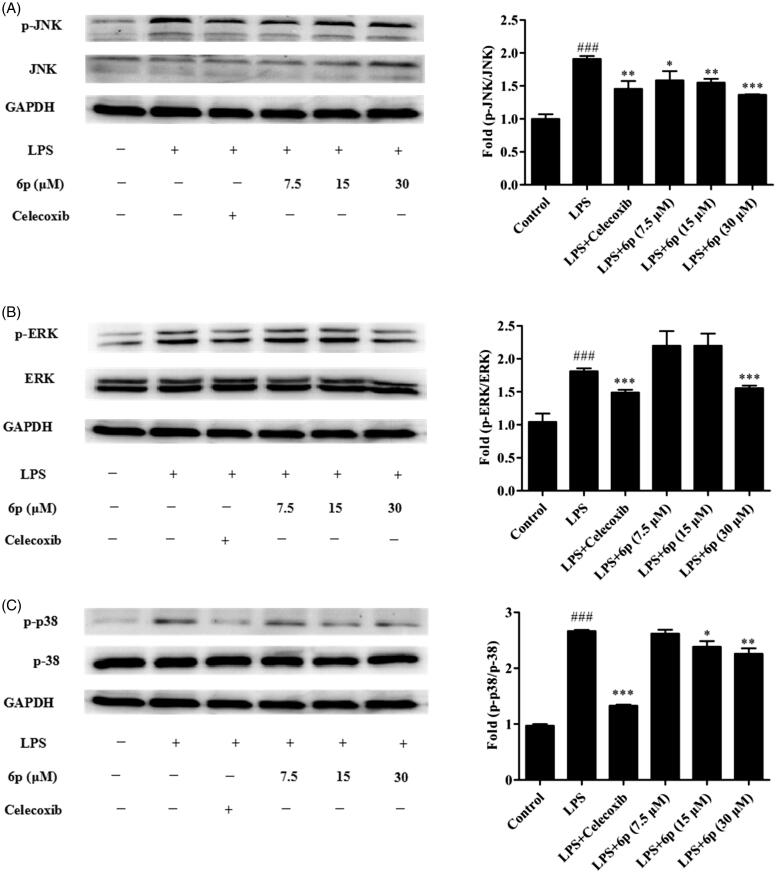
Compound **6p** inhibited LPS-induced inflammatory response in RAW 264.7 cells. Cells were pre-treated with different concentrations of **6p** (7.5–30 µM) and Celecoxib (7.5 µM) for 24 h. The levels of p-p38/P38, p-ERK/ERK, p-JNK/JNK and GAPDH proteins, and their phosphorylated forms were analysed using Western blotting. Total cellular proteins were prepared and analysed by Western blotting. Results are the mean ± SD, *n* = 3. ### *p* < .001, vs. Control. *p < .05, **p < .01, ***p < .001 vs. LPS alone.

As upstream proteins, phosphorylation of the MAPK family of proteins is closely related to and plays an important role in the expression of iNOS and COX-2, and the release of inflammatory factors in LPS-induced inflammatory responses^13^^,^^14^. To find the molecular target of compound **6p** further upstream of the MAPK signalling pathway, the effects of compound **6p** on the LPS-induced phosphorylation of ERK, JNK, and p38 MAPKs in RAW 264.7 cells were assessed by western blotting. Celecoxib (7.5 µM) was used as positive control. As expected (Figure 4), phosphorylation of p38, JNK, and ERK increased after LPS stimulation. Compound **6p** markedly diminished the phosphorylation of JNK (*p* < .001), ERK (*p* < .001) and p38 (*p* < .01) MAPKs at 30 µM (Figure 4(A–C)). Furthermore, compound **6p** (7.5, 15, and 30 µM) inhibited the LPS-induced phosphorylation of JNK and ERK in a concentration-dependent manner. These results suggest that the anti-inflammatory effect of compound **6p** might be associated with the inhibitory effects on the MAPK signalling pathways.

### 3.7. In vivo anti-inflammatory activity

**Figure 5.**
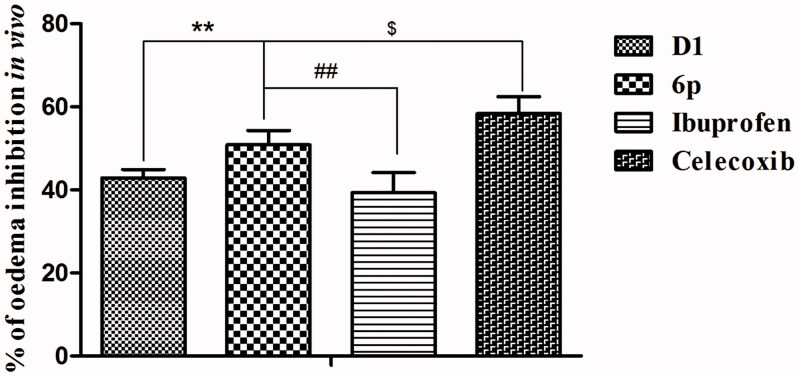
The inhibited paw swelling actives of lead compound **D1**, **6p** and positive control drugs (25 mg/kg) in a carrageenan-induced mouse paw oedema model after 3 h of oral administration. **p < .01, vs. **D1**; ##*p* < .01, vs. Ibuprofen; $ *p* < .05, vs. Celecoxib.

To evaluate the anti-inflammatory activity of compound **6p** *in vivo*, a carrageenan-induced mouse paw oedema model was used^10^^,^^26^. Each test compound (lead compound **D1, 6p,** and the positive controls ibuprofen and celecoxib) was administered orally at a dose of 25 mg/kg body weight prior to the induction of inflammation by carrageenan (0.1 ml) injection. The paw volume was measured over 5 h (at 0, 1, 3, and 5 h) and the percent inhibition of oedema was calculated (Table 2). Inhibition of paw swelling was the highest in mice 3 h after carrageenan administration by all the tested compounds. It is noteworthy that compound **6p** displayed anti-inflammatory effects (50.83%) that were significantly greater than those of the lead compound **D1** (42.81%, *p* < .01) and ibuprofen (39.30%, *p* < .01), but slightly lesser than those of celecoxib (58.34%, *p* < .05) (Figure 5).

**Table 2.** The anti-inflammatory activity and ulcerogenic activity *in vivo*.

Compounds	% of oedema inhibition (% mean ± SD)^a^			% ulceration
	1 h	3 h	5 h	
**D1**	38.26 ± 2.82	42.81 ± 2.11	28.71 ± 2.92	20
**6p**	37.01 ± 8.45	50.83 ± 3.47	38.24 ± 6.04	20
**Ibuprofen**	31.92 ± 5.76	39.30 ± 4.86	25.29 ± 3.43	100
**Celecoxib**	48.09 ± 5.33	58.34 ± 4.08	41.92 ± 2.05	0

^a^ Values are expressed as the mean ± S.D (*n* = 4).

Control: 0.5% sodium CMC solution in distilled water (25 ml/kg, *p.o.*).

Compounds and positive drugs were administered at a dose of 25 mg/kg, *p.o*. in 0.5% sodium CMC solution.

### 3.8. Ulcerogenic effect

In order to evaluate the damage of the synthesised compound to the gastric mucosa, acute ulcerogenic studies were carried out for the most biologically active synthesised compound **6p**, lead compound **D1**, ibuprofen and celecoxib as standard was evaluated using the reference reported procedure^27^. Results are presented in Table 2 as % ulceration. The ulceration effect of compound **6p** was stronger than the selective COX-2 inhibitor celecoxib (no ulceration), which was weaker than the non-selective COX-2 inhibitor ibuprofen that showed 100% ulceration.

## 4. Conclusions

In summary, we designed, synthesised, and characterised a series of 5-alkyl-4-oxo-4,5-dihydro-[1, 2, 4]triazolo[4,3-a]quinoxaline-1-carboxamide derivatives using by ^1^H-NMR, ^13 ^C-NMR and HRMS. All the tested compounds inhibited LPS-induced NO production in RAW264.7 macrophages. Among them, compound **6p** exhibited the most significant activity and was more potent than the lead compound **D1**. Investigation of the mechanisms underlying the anti-inflammatory activity of compound **6p** showed that it inhibits the expression of iNOS and COX-2 and the production of NO, TNF-α, and IL-6 by suppressing the LPS-induced MAPK signalling pathway.

Furthermore, compound **6p** displayed more prominent anti-inflammatory activity and weaker ulceration than the positive control ibuprofen in the *in vivo* acute inflammatory model. These findings indicate that compound **6p** could serve as a promising anti-inflammatory agent and warrants further studies.

## Supplementary Material

Supplemental Material
