# Supplementary material for: Discovery and evaluation of novel synthetic 5-alkyl-4-oxo-4,5-dihydro-[1,2,4]triazolo[4,3-a]quinoxaline-1-carbox-amide derivatives as anti-inflammatory agents
Source: J Enzyme Inhib Med Chem. 2019 Nov 11;35(1):85–95. doi: 10.1080/14756366.2019.1680658 (PMC6853232; doi:10.1080/14756366.2019.1680658)
Supplement: Supplemental Material [file IENZ_A_1680658_SM1222.pdf]

Discovery and evaluation of novel synthetic 5-alkyl-4-oxo-4,5-dihydro-[1,2,4]triazolo[4,3-a]quinoxaline-1-carbox-amide derivatives as anti-inflammatory agents

^{1}H NMR , ^{13}C NMR and HRMS spectra of target compounds

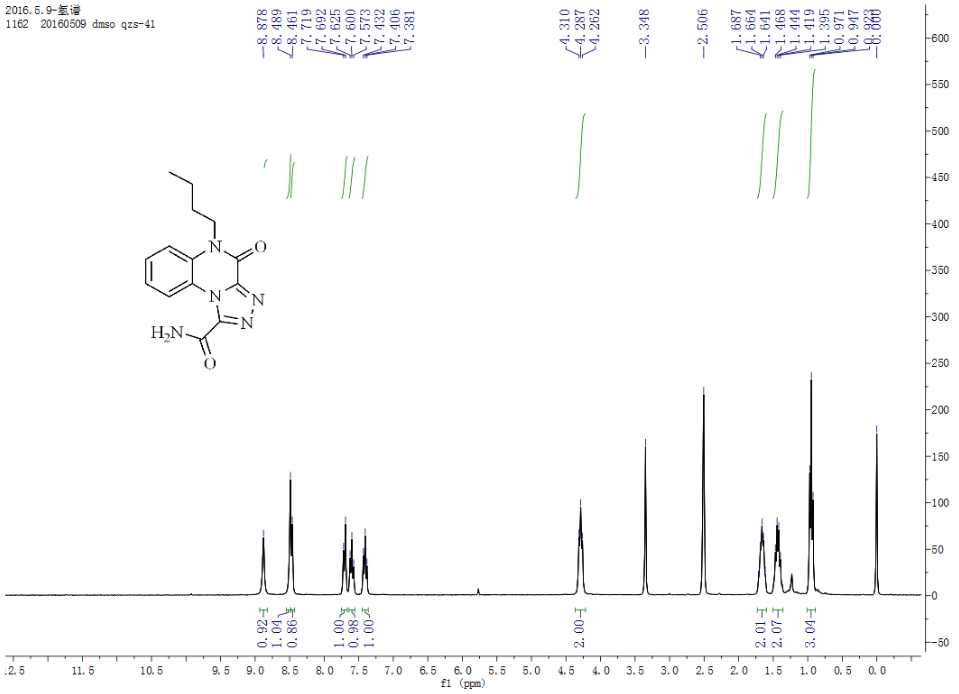

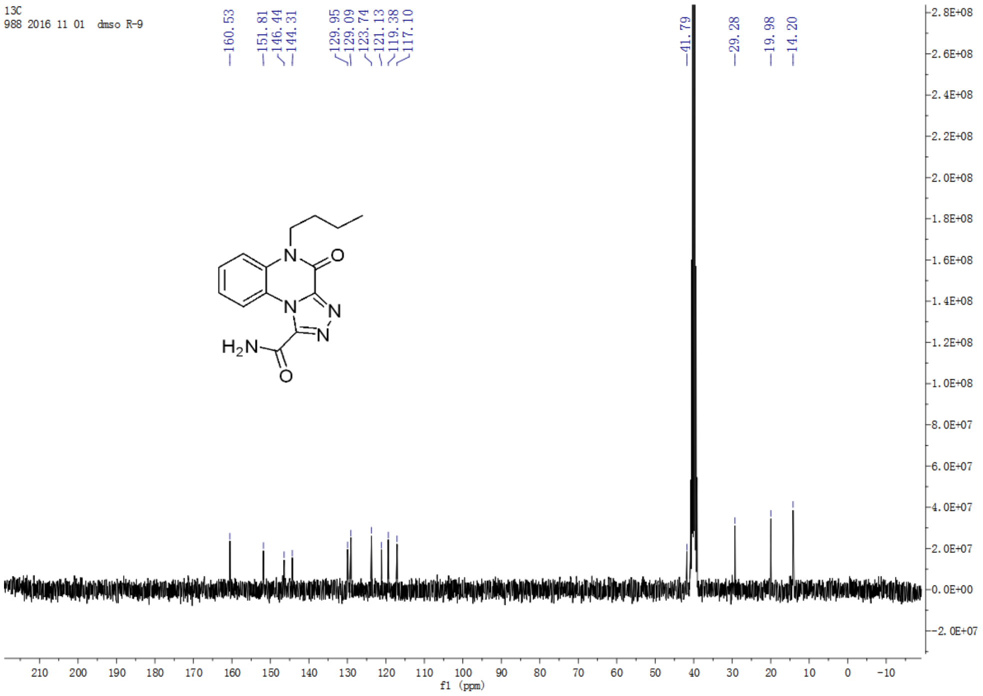

^{1}H NMR and ^{13}C NMR spectra of target compound ***6a***

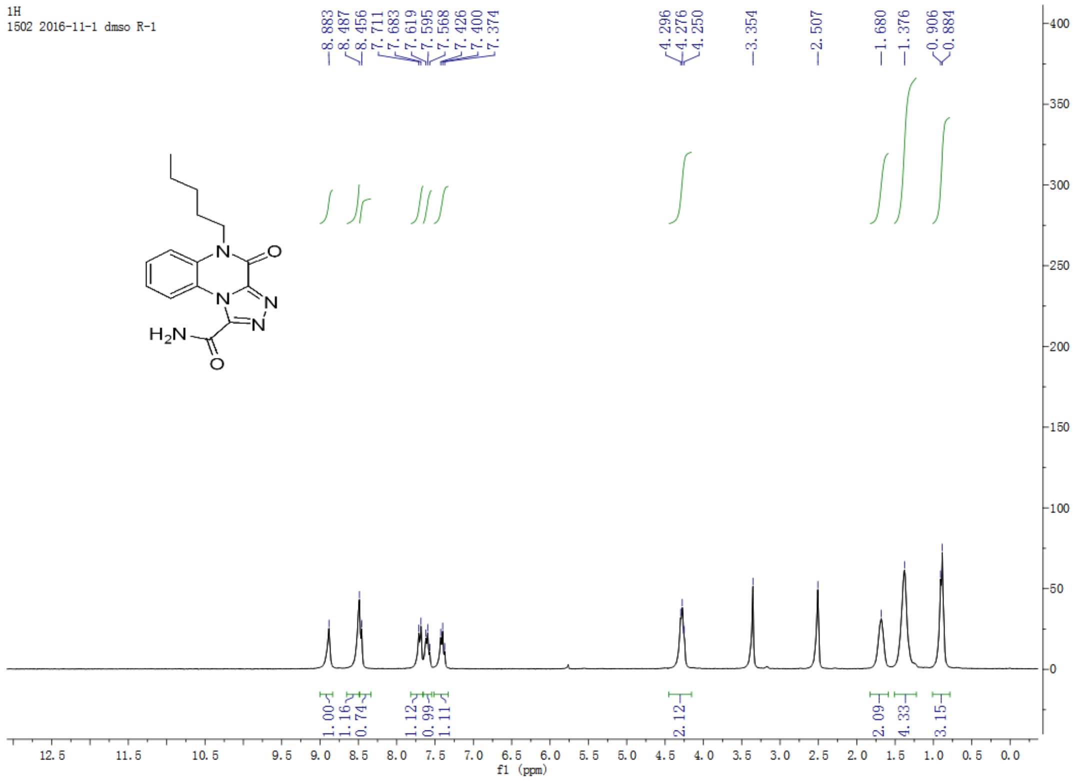

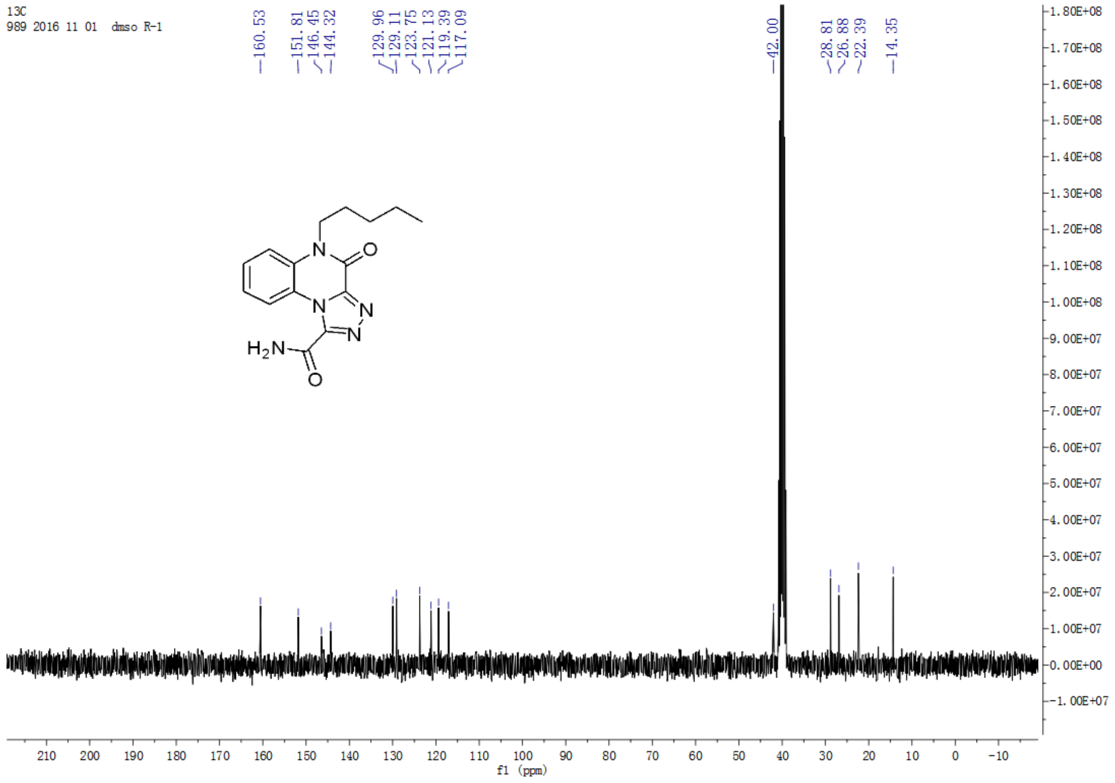

^{1}H NMR and ^{13}C NMR spectra of target compound **6b**

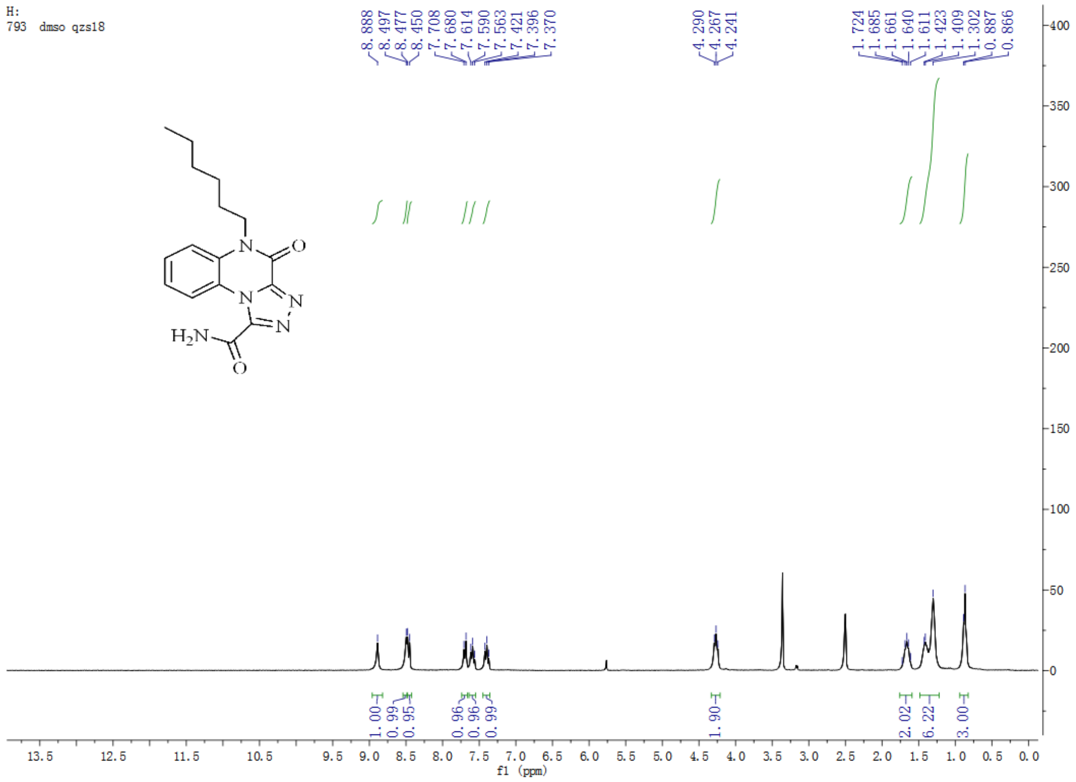

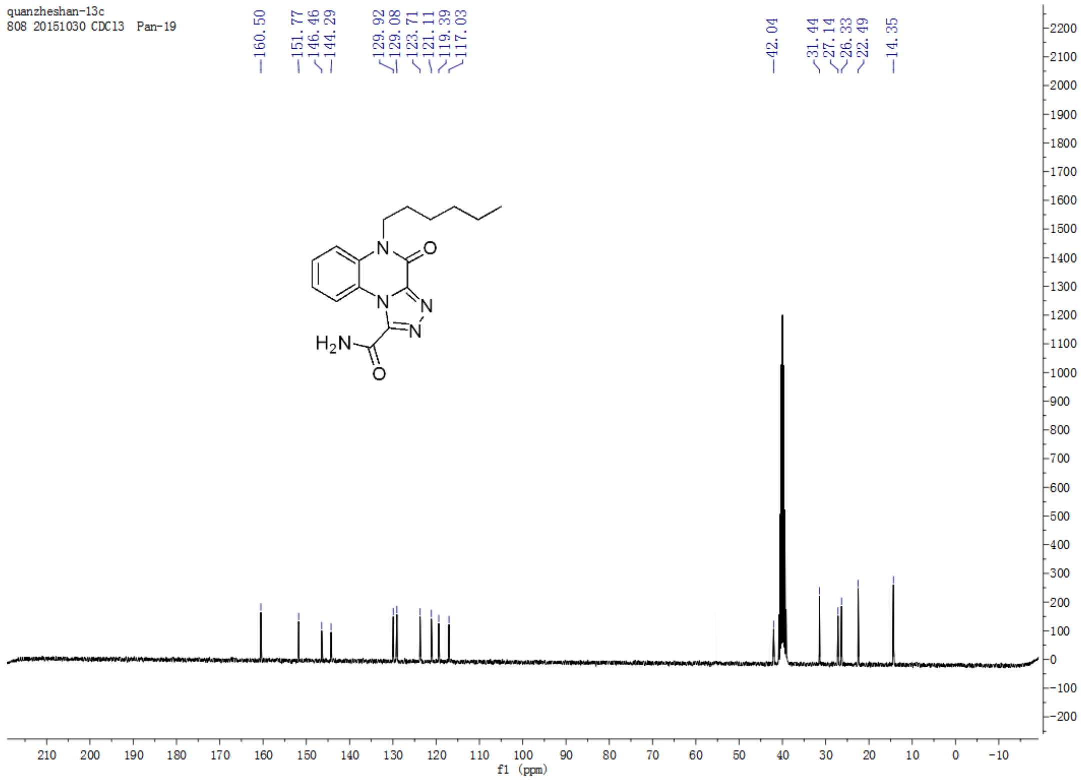

^{1}H NMR and ^{13}C NMR spectra of target compound **6c**

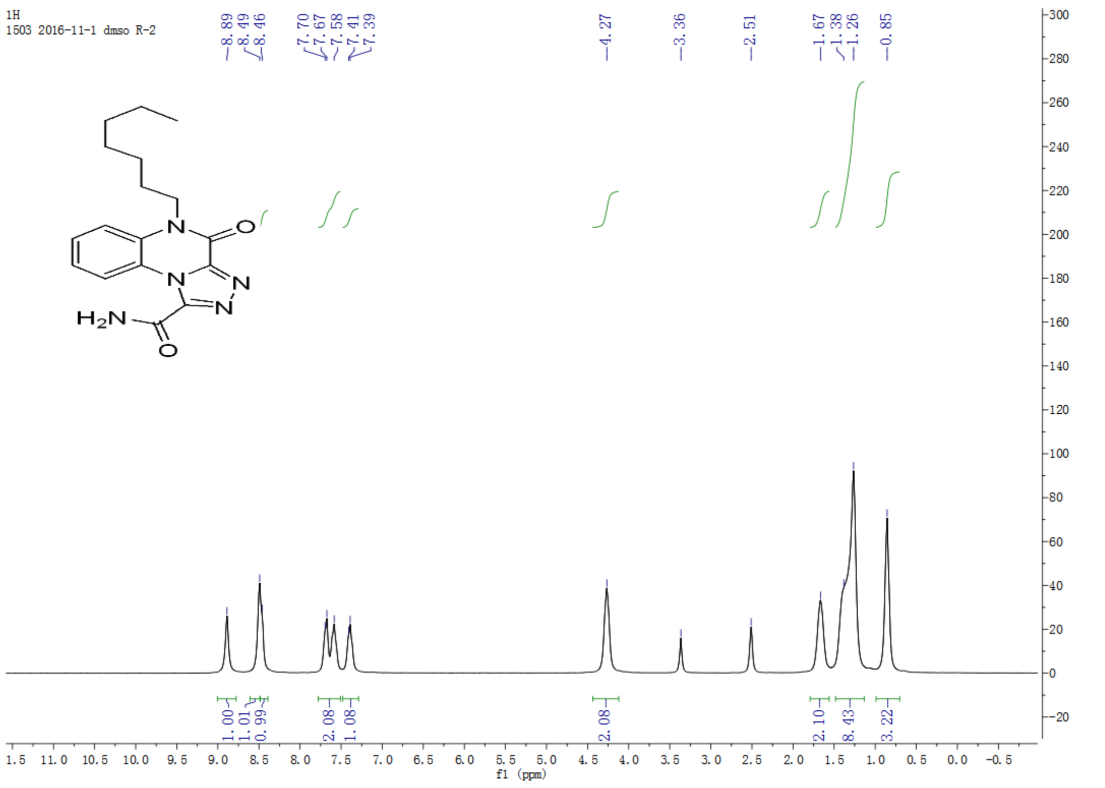

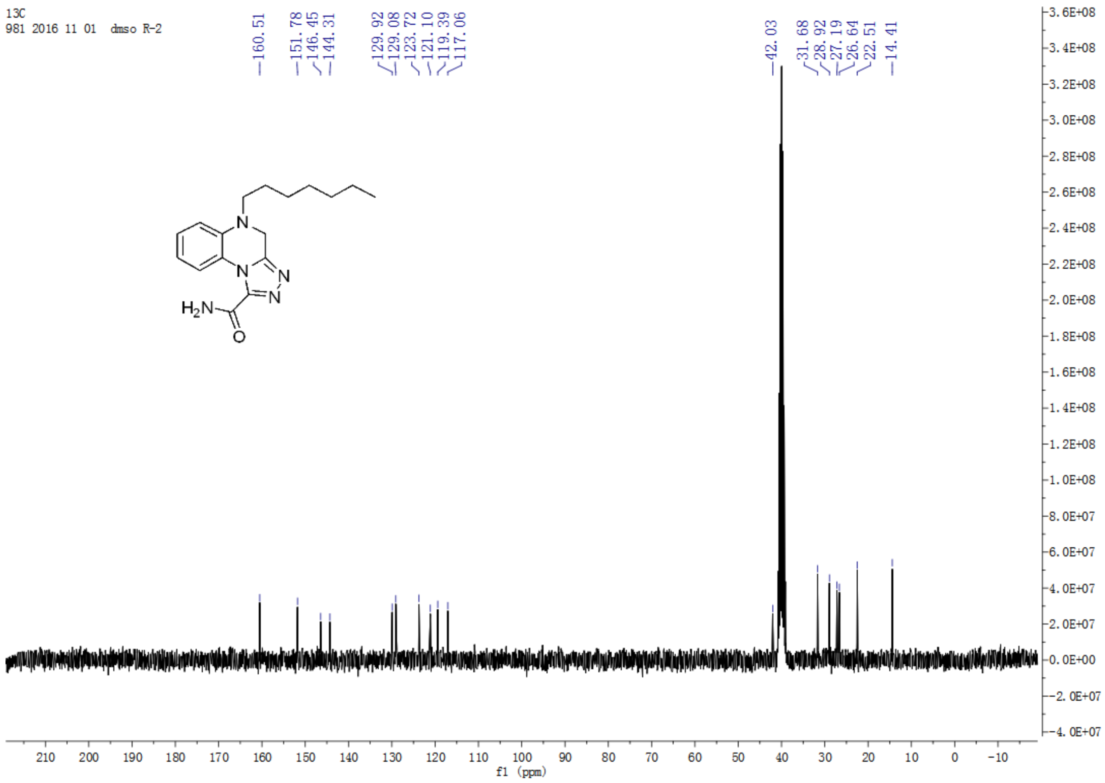

^{1}H NMR and ^{13}C NMR spectra of target compound **6d**

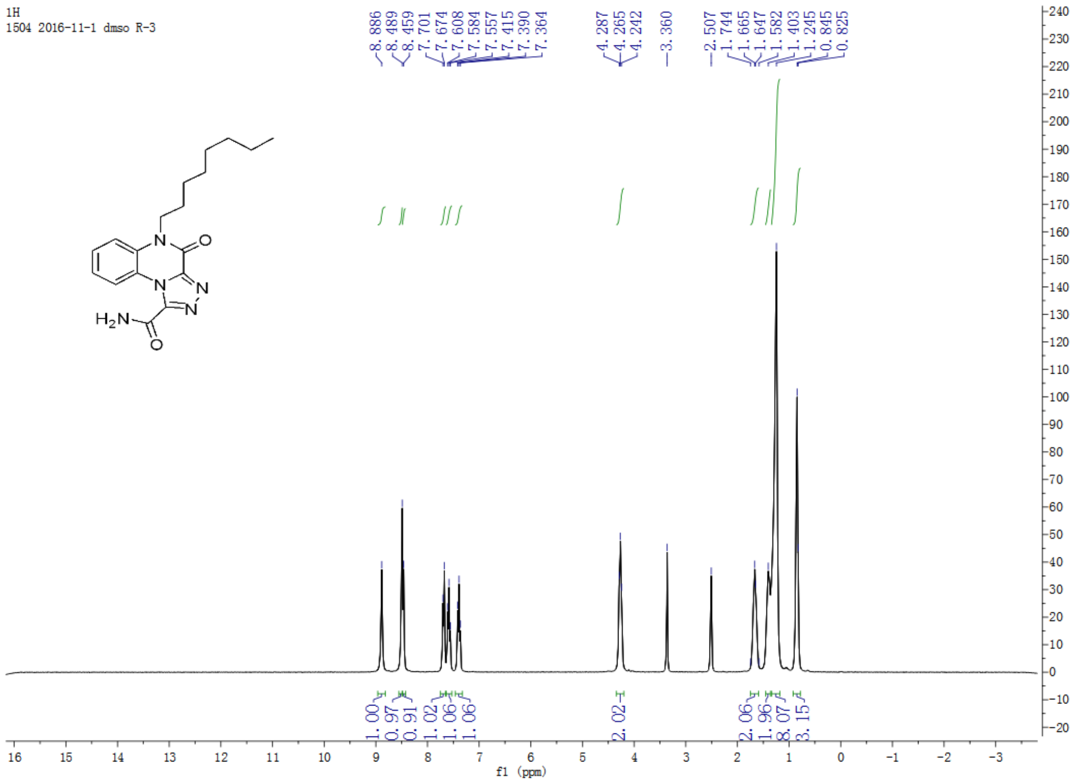

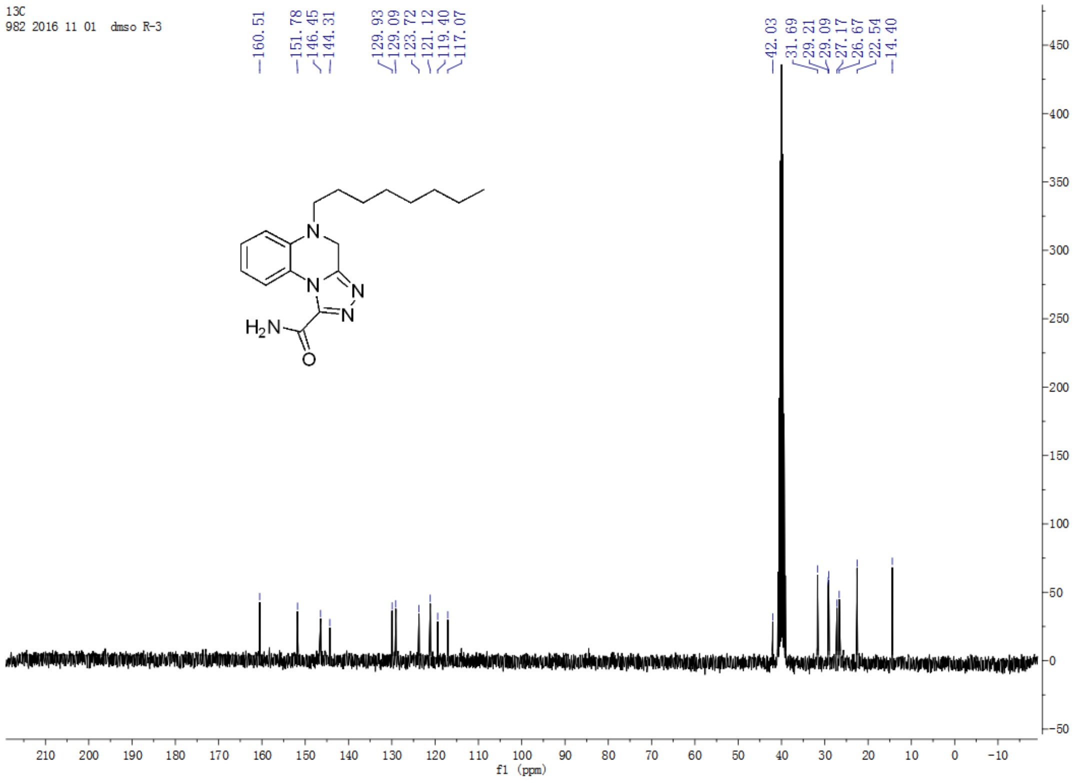

*^{1}H NMR and ^{13}C NMR spectra of target compound **6e***

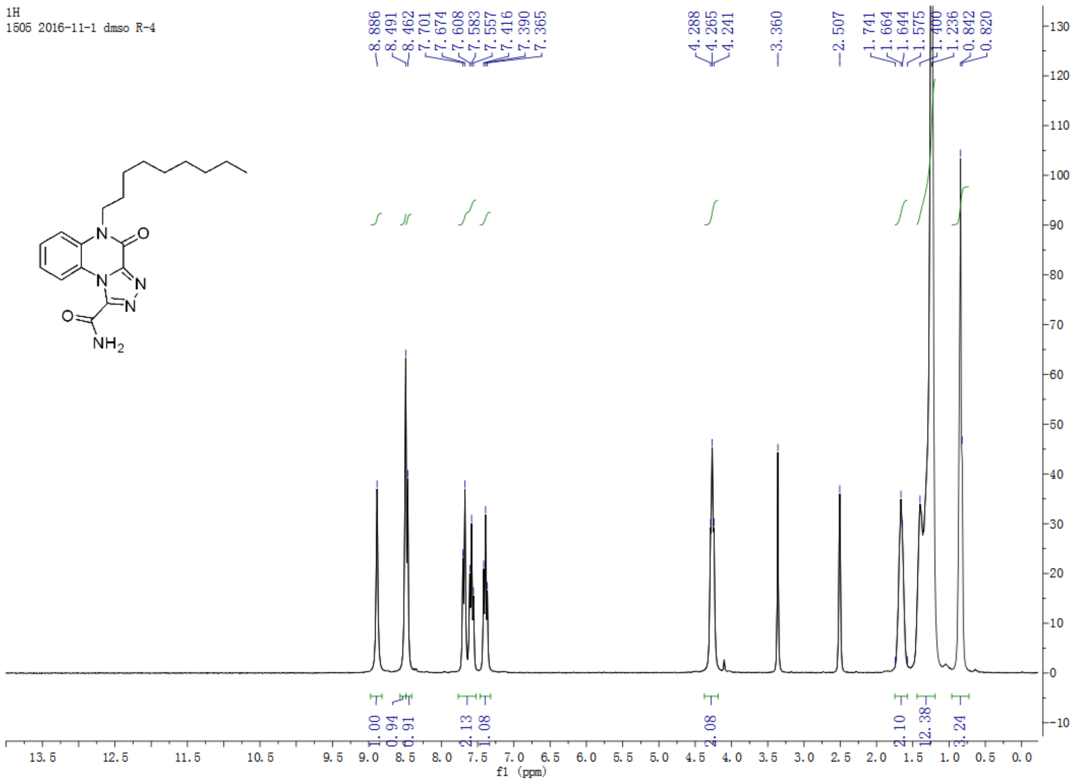

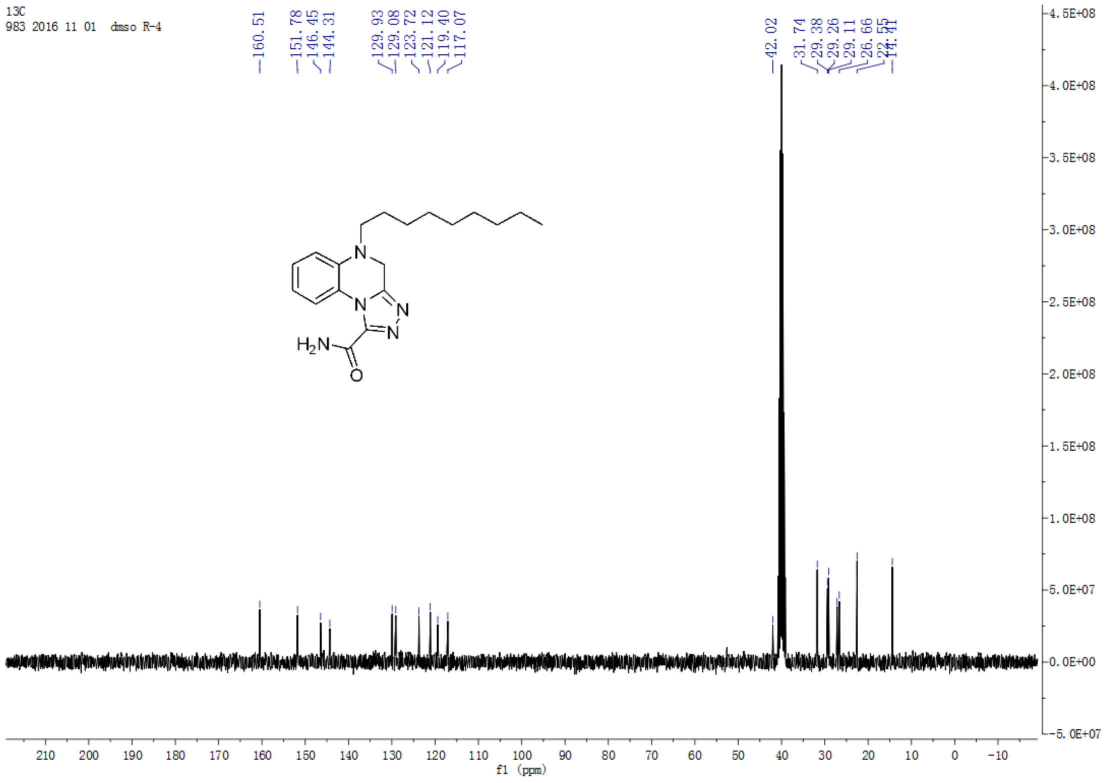

^{1}H NMR and ^{13}C NMR spectra of target compound ***6f***

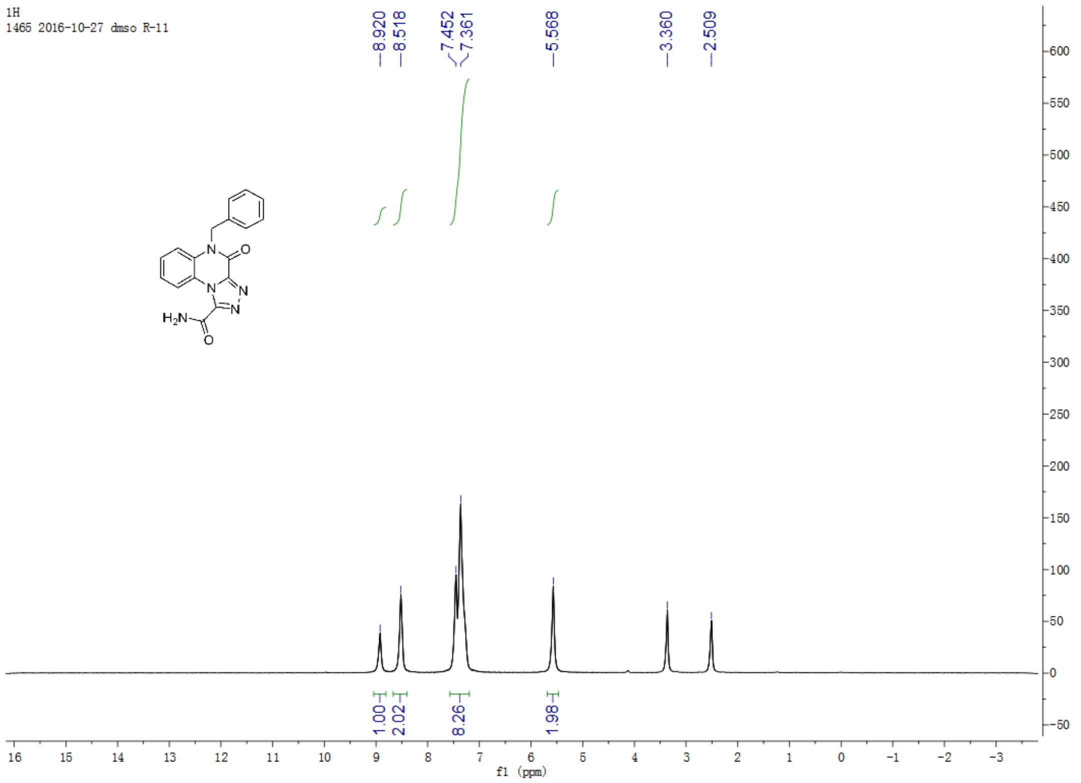

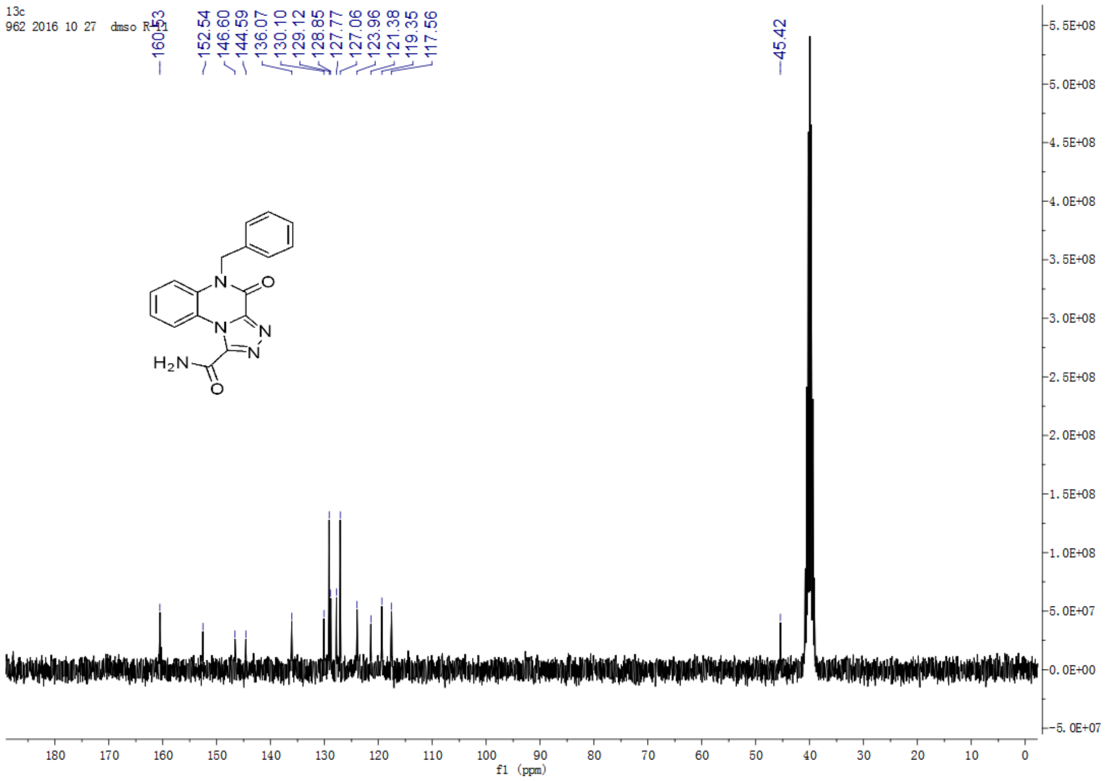

^{1}*H NMR and* ^{13}*C NMR spectra of target compound* ***6g***

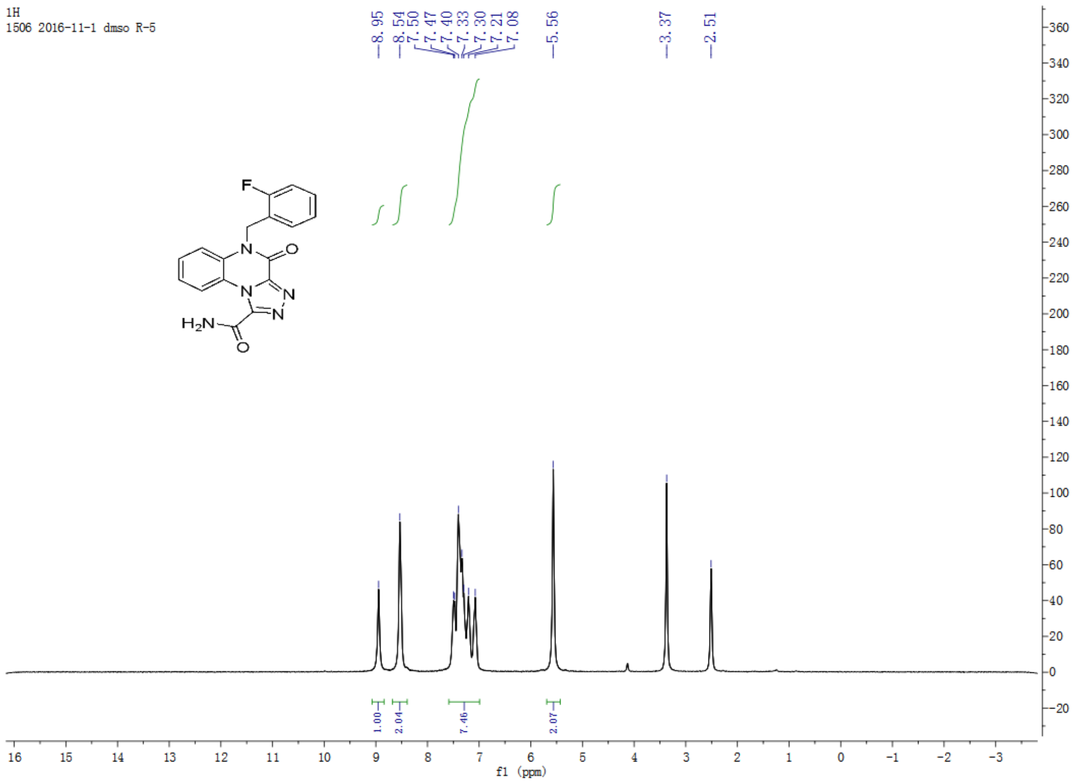

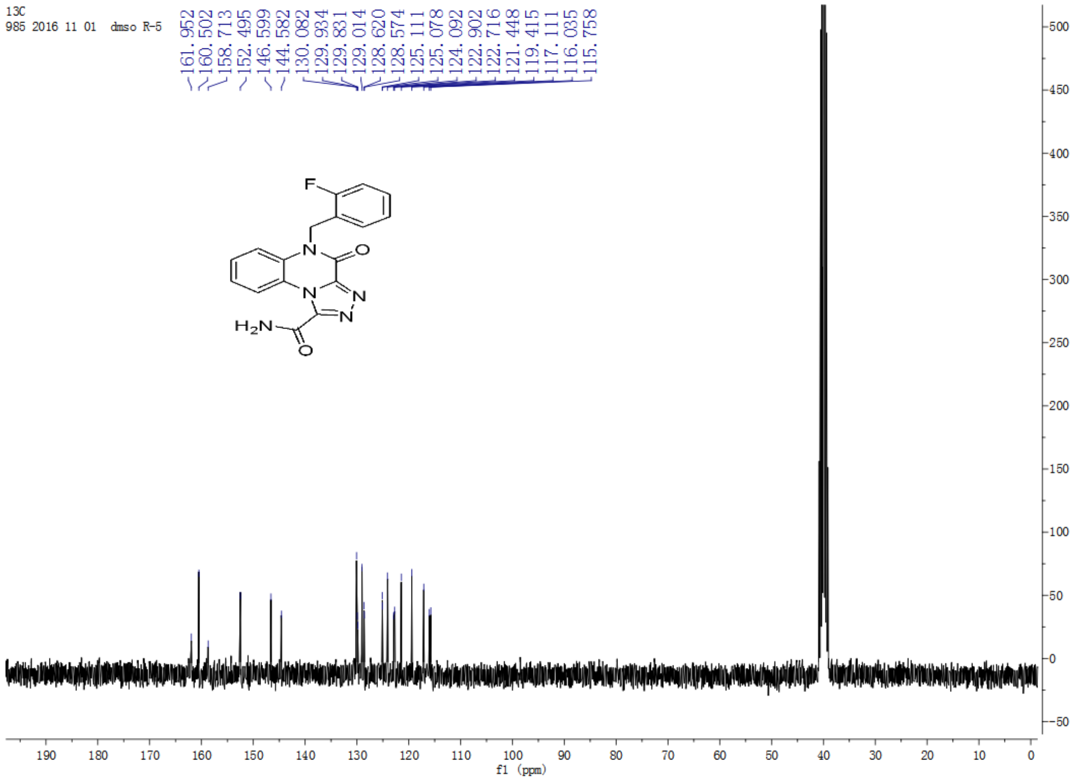

^{1}H NMR and ^{13}C NMR spectra of target compound ***6h***

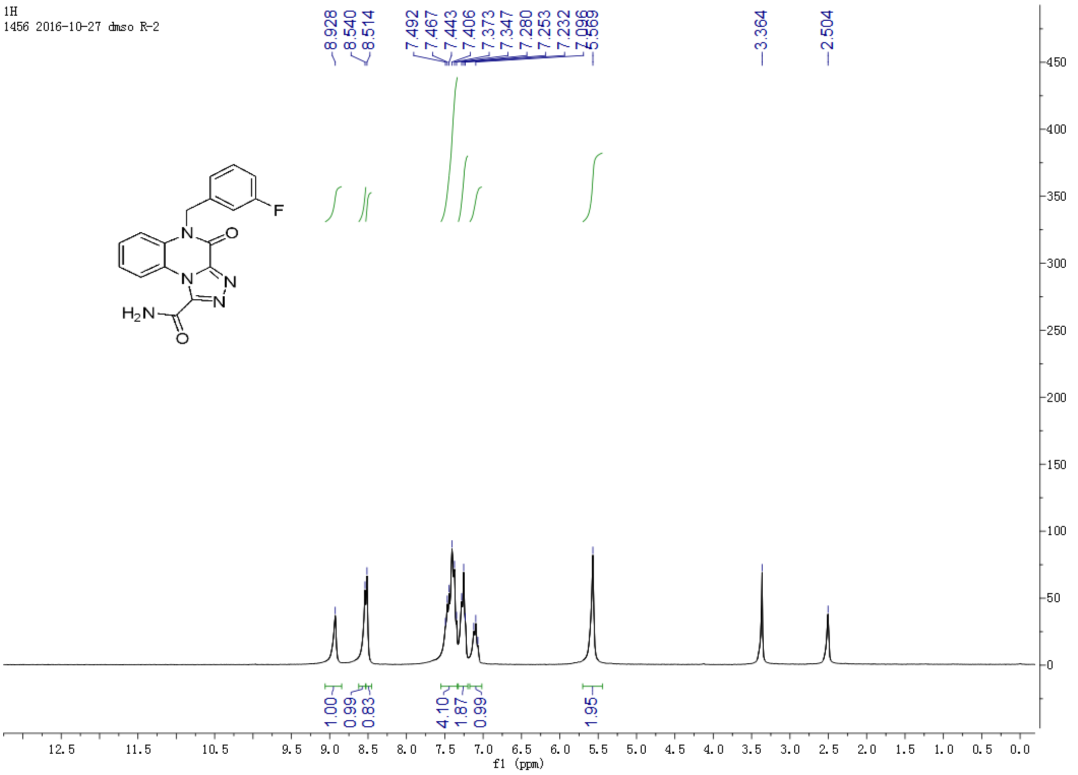

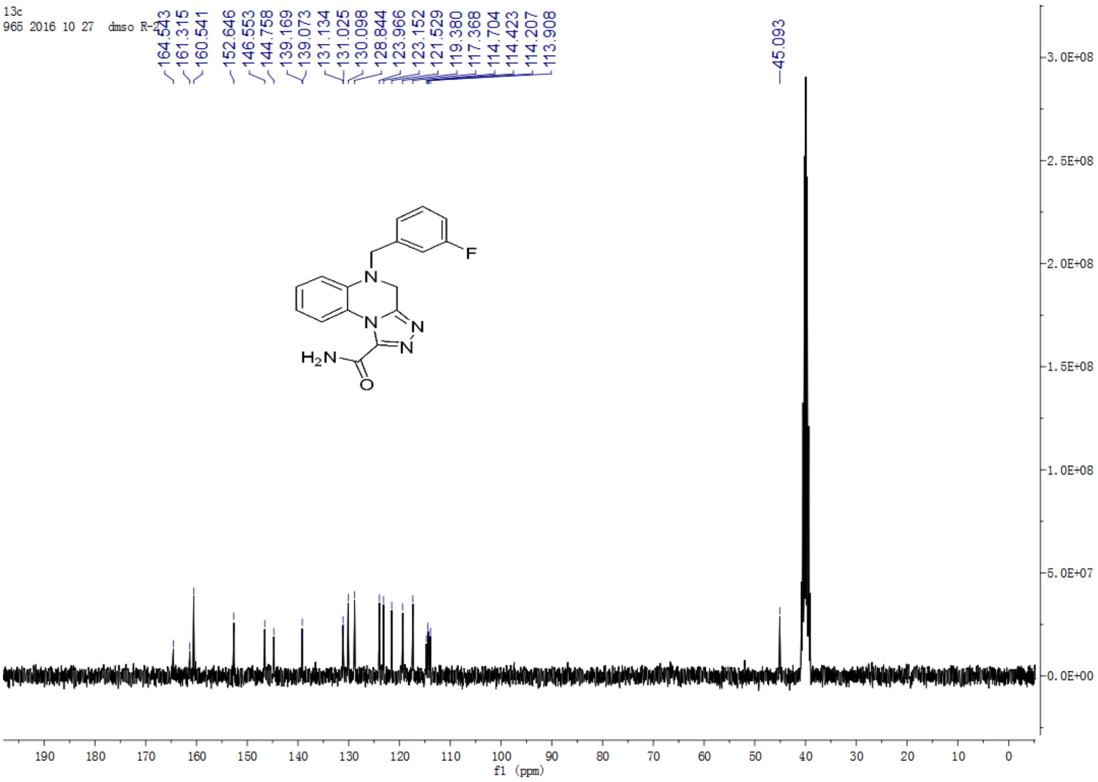

^{1}*H NMR and* ^{13}*C NMR spectra of target compound* ***6i***

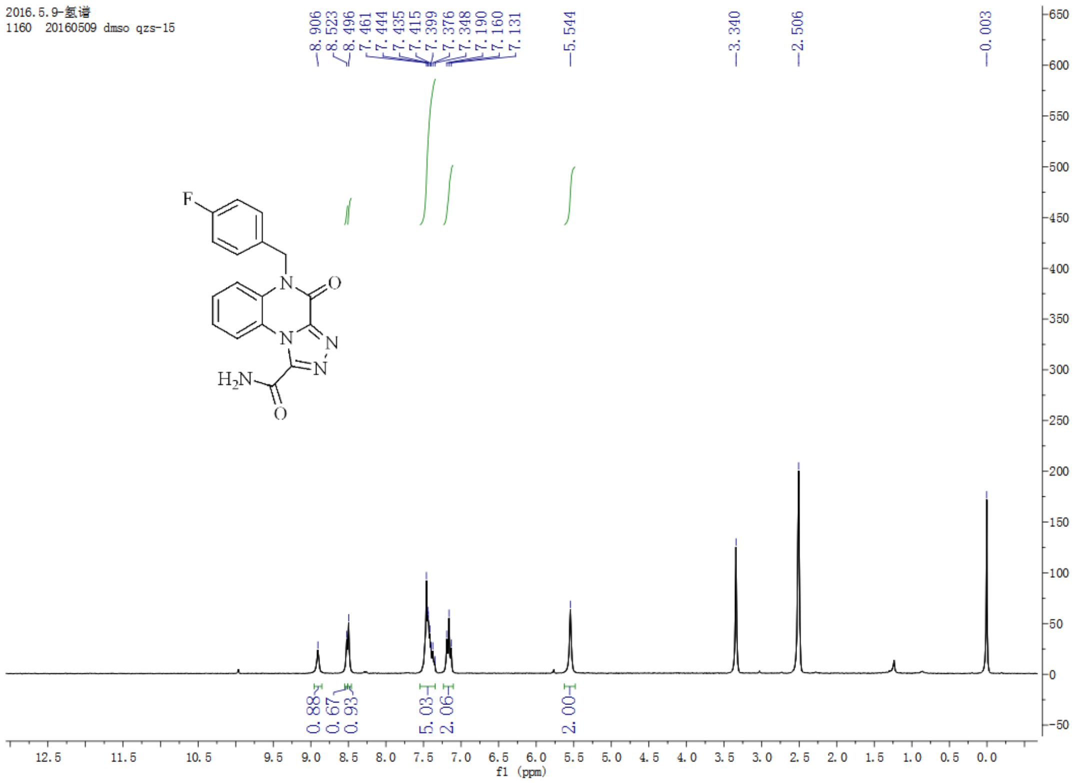

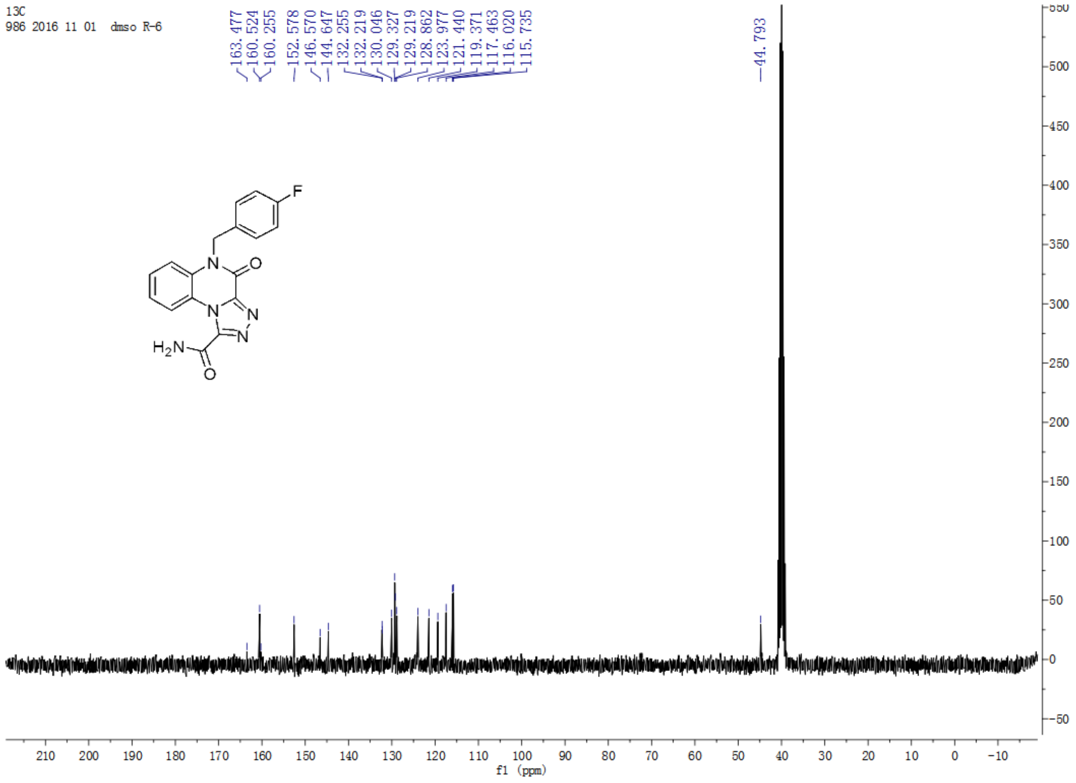

^{1}H NMR and ^{13}C NMR spectra of target compound ***6j***

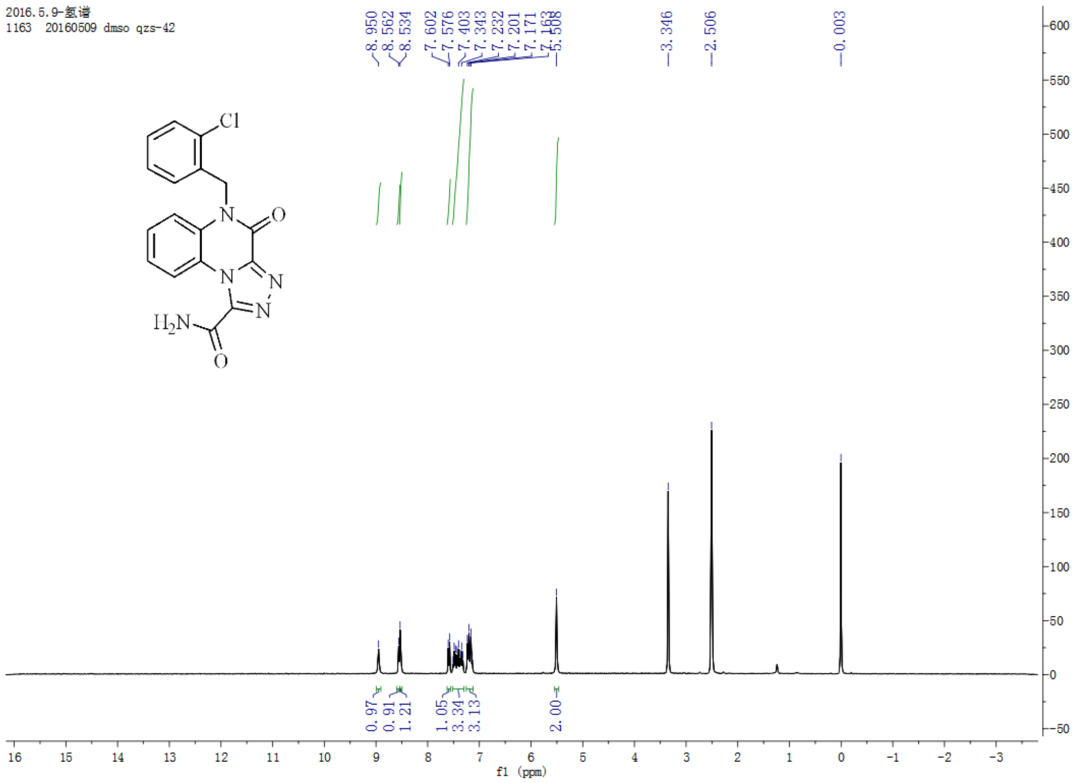

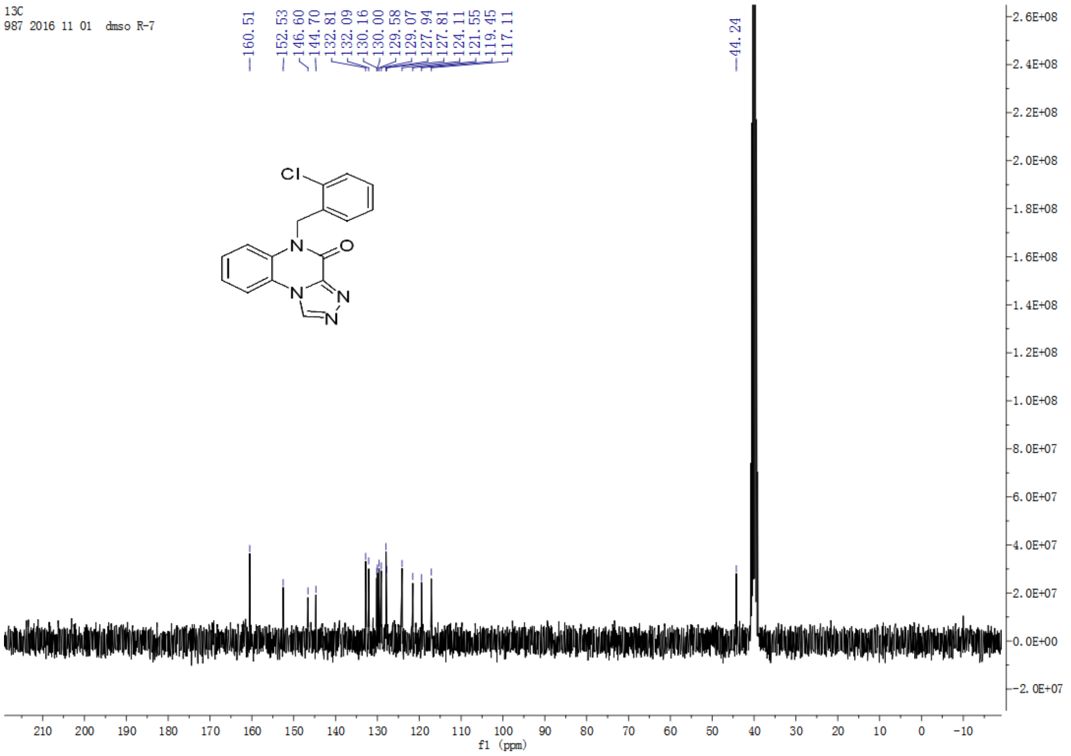

*^{1}H NMR and ^{13}C NMR spectra of target compound **6k***

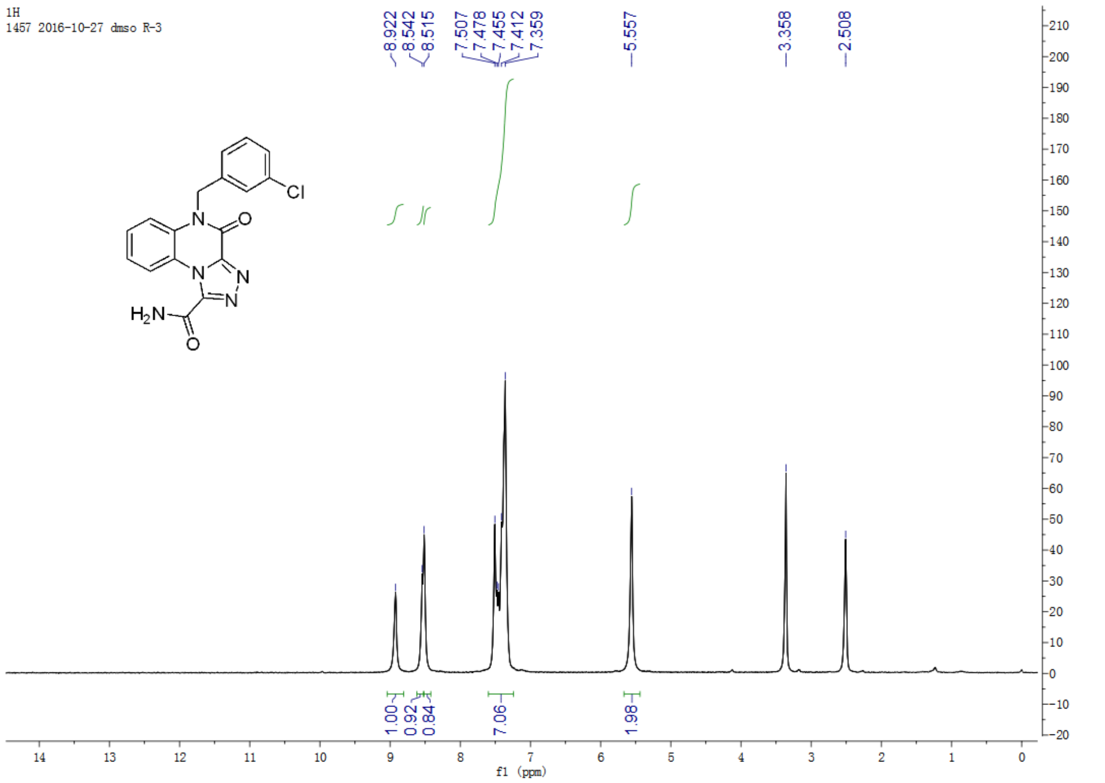

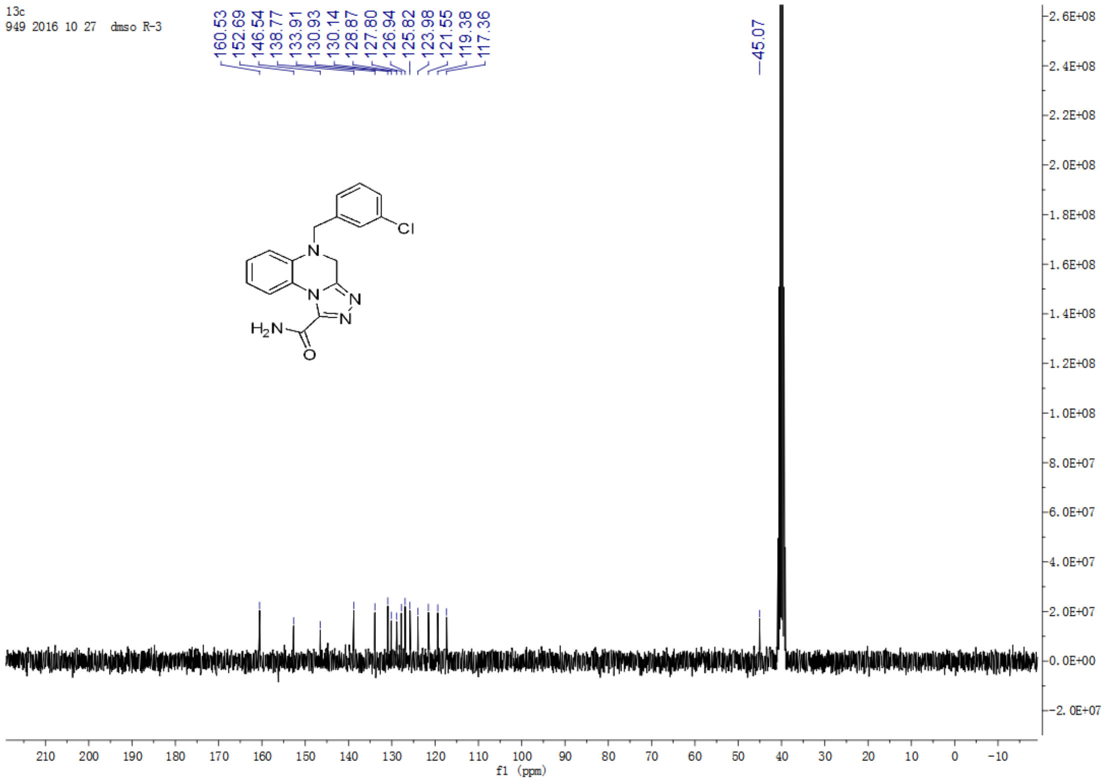

^{1}H NMR and ^{13}C NMR spectra of target compound ***6l***

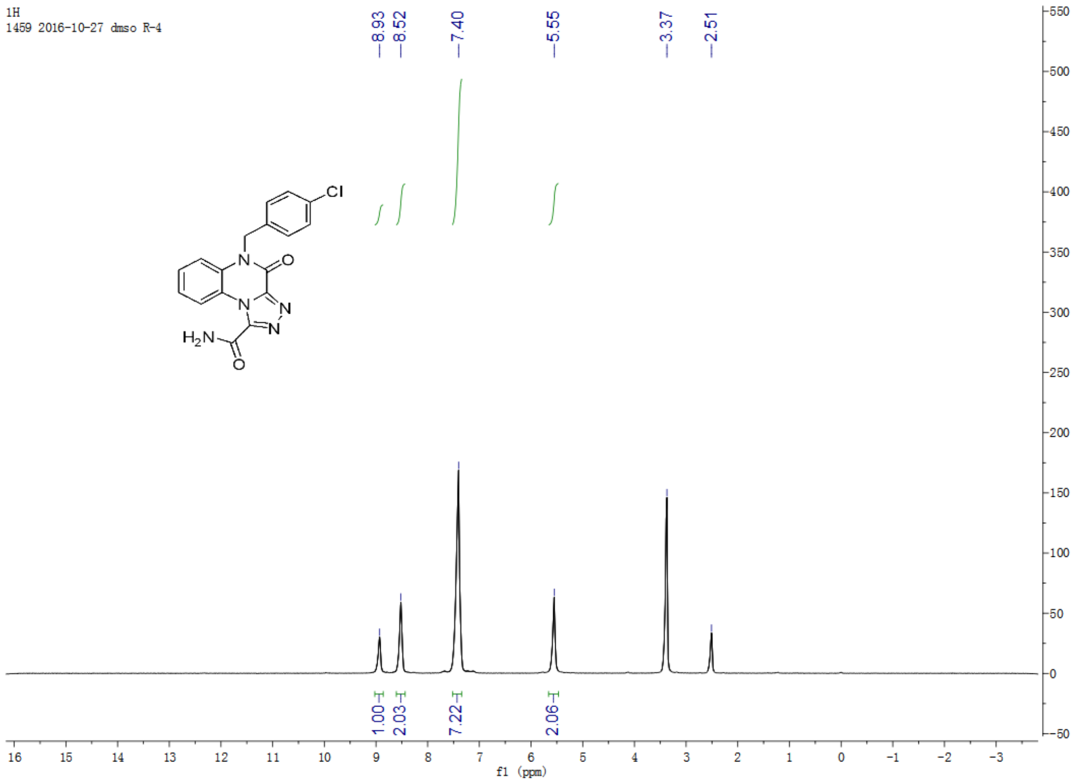

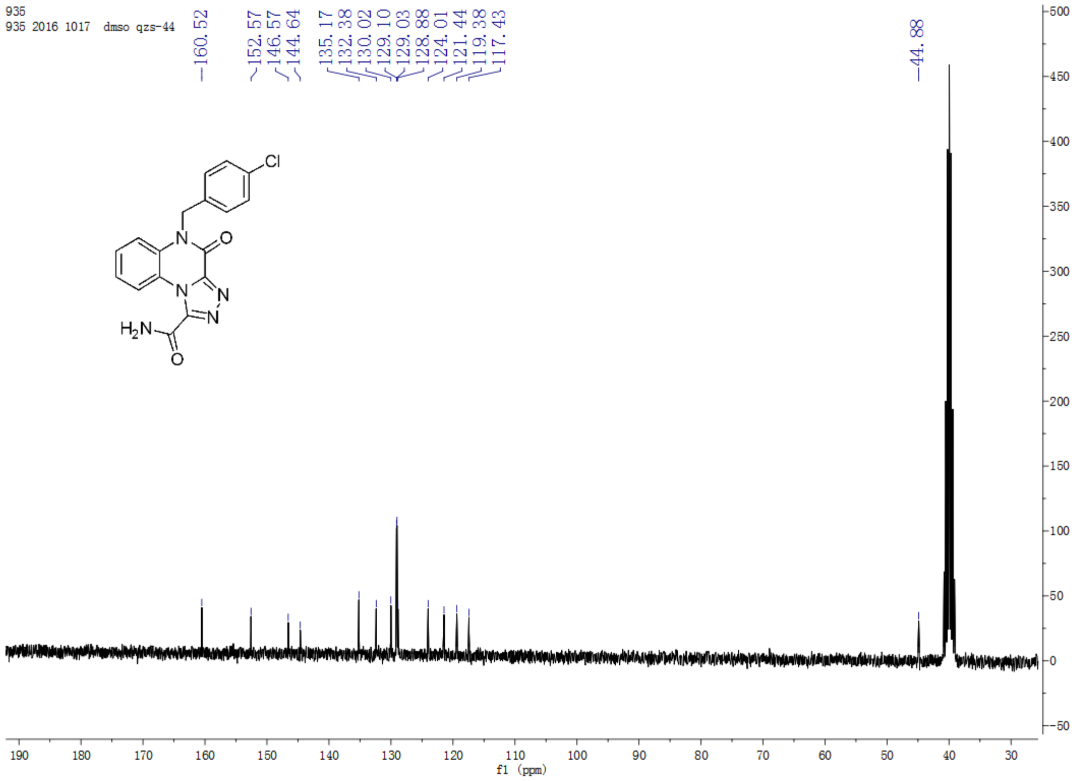

^{1}H NMR and ^{13}C NMR spectra of target compound ***6m***

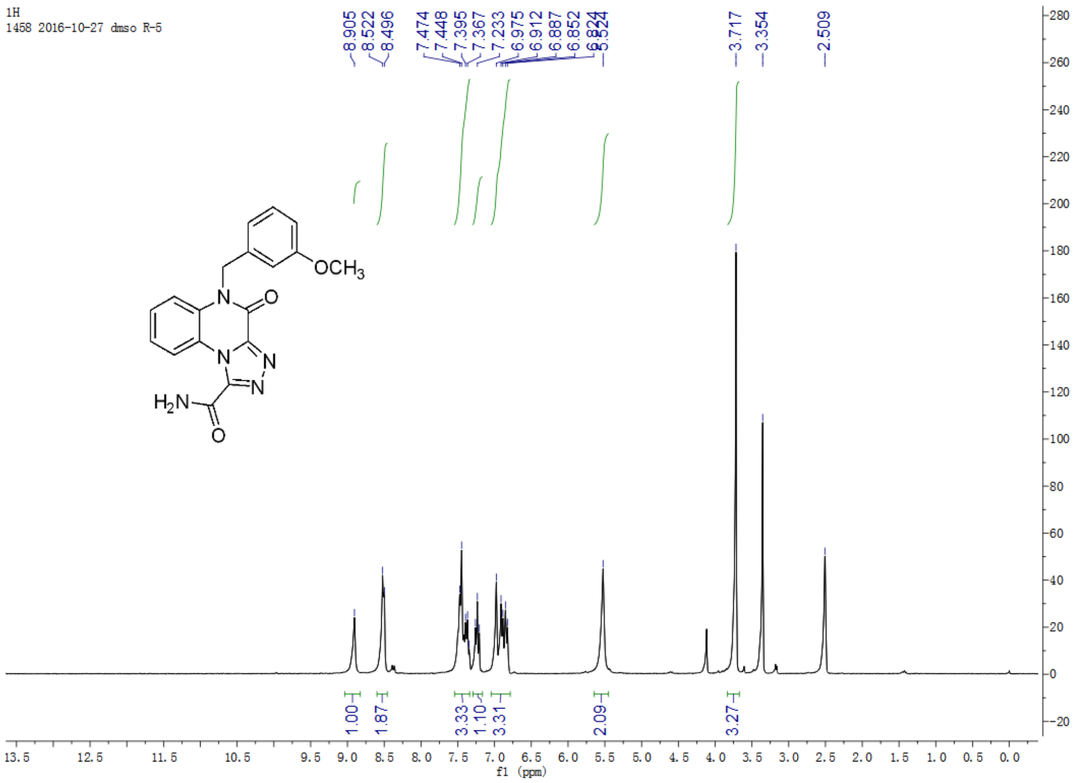

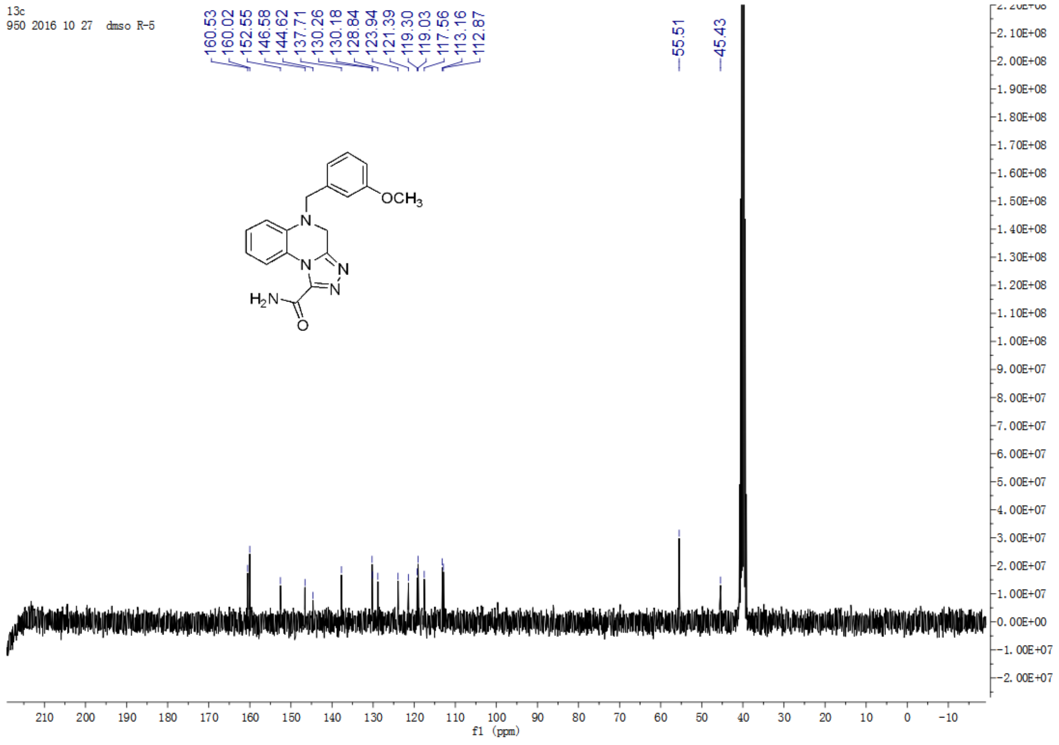

^{1}H NMR and ^{13}C NMR spectra of target compound ***6n***

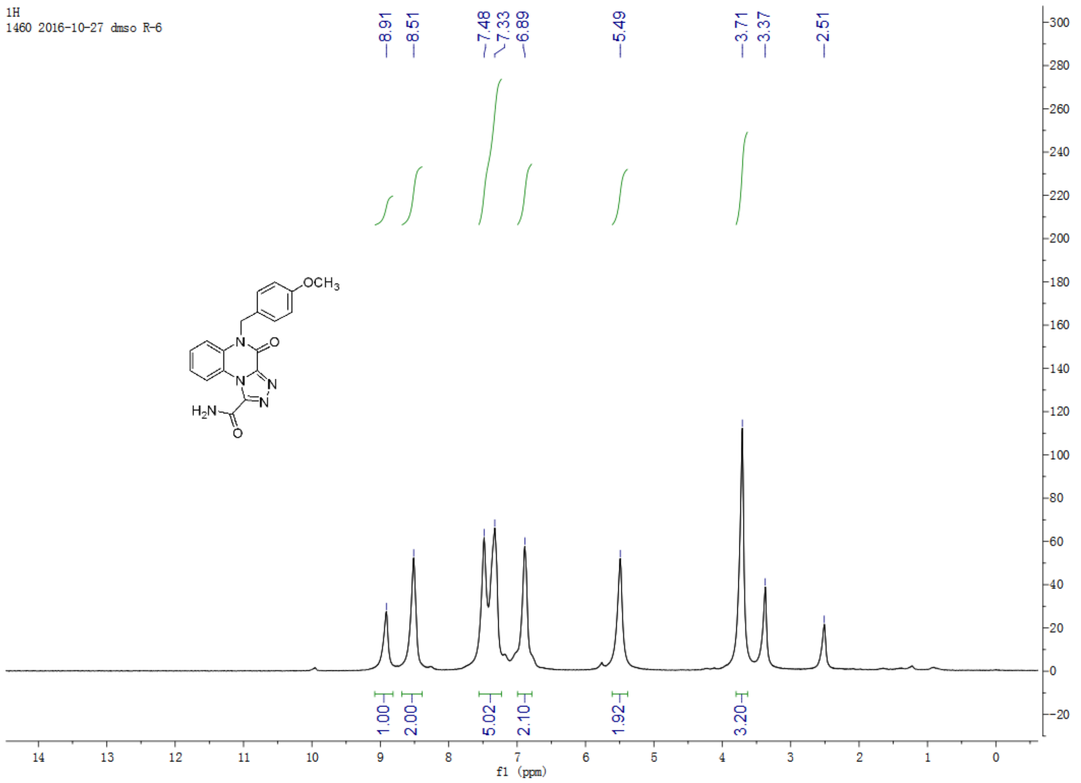

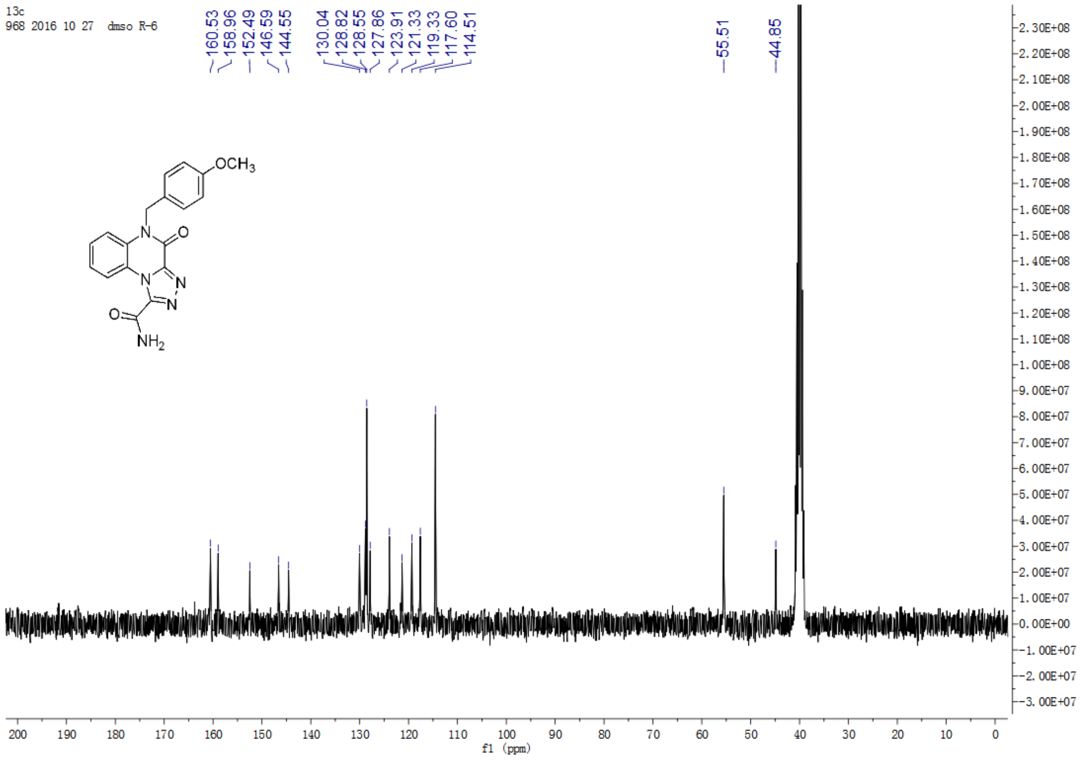

^{1}H NMR and ^{13}C NMR spectra of target compound ***6o***

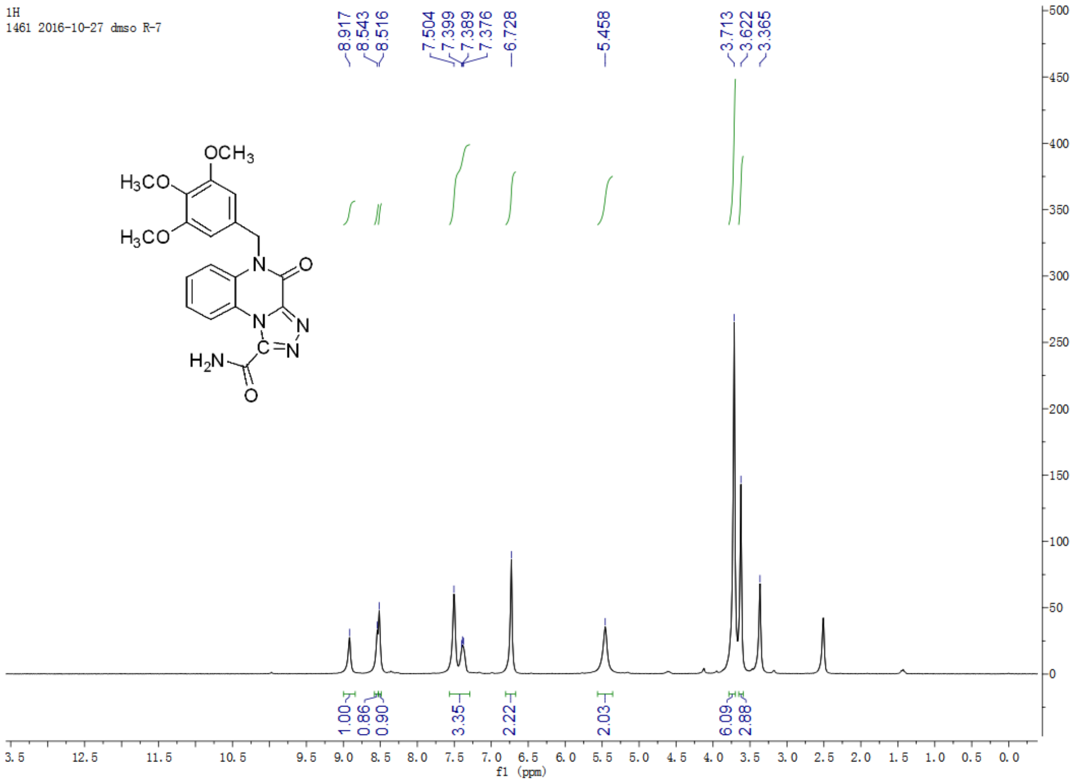

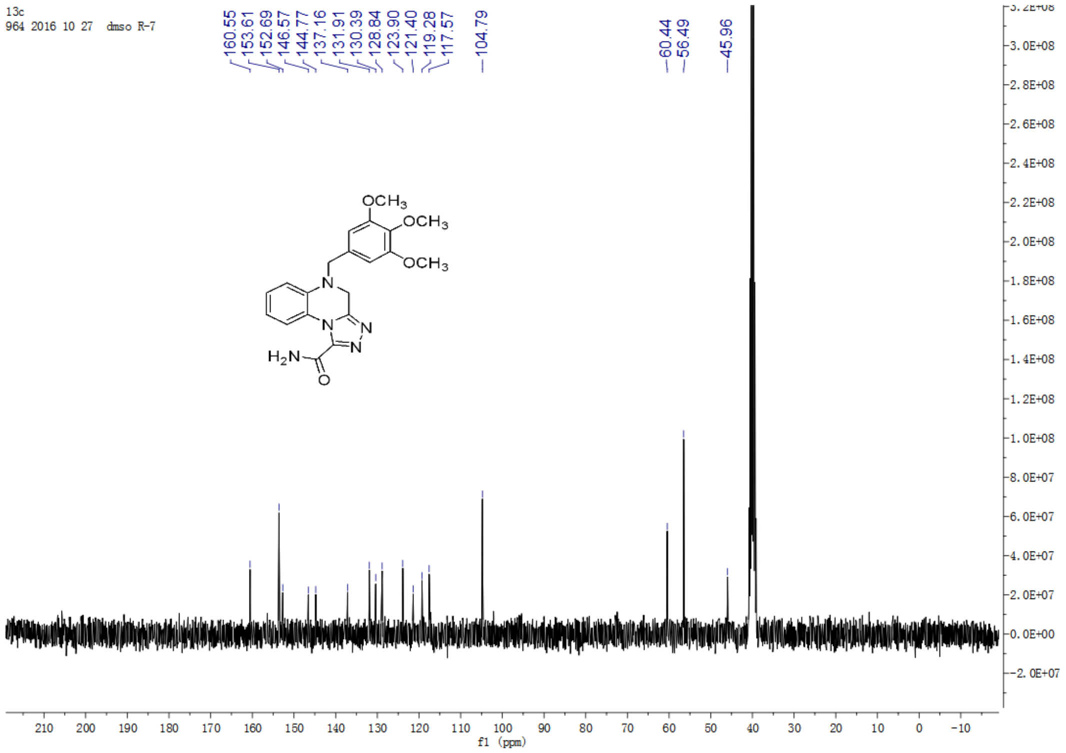

^{1}H NMR and ^{13}C NMR spectra of target compound ***6p***

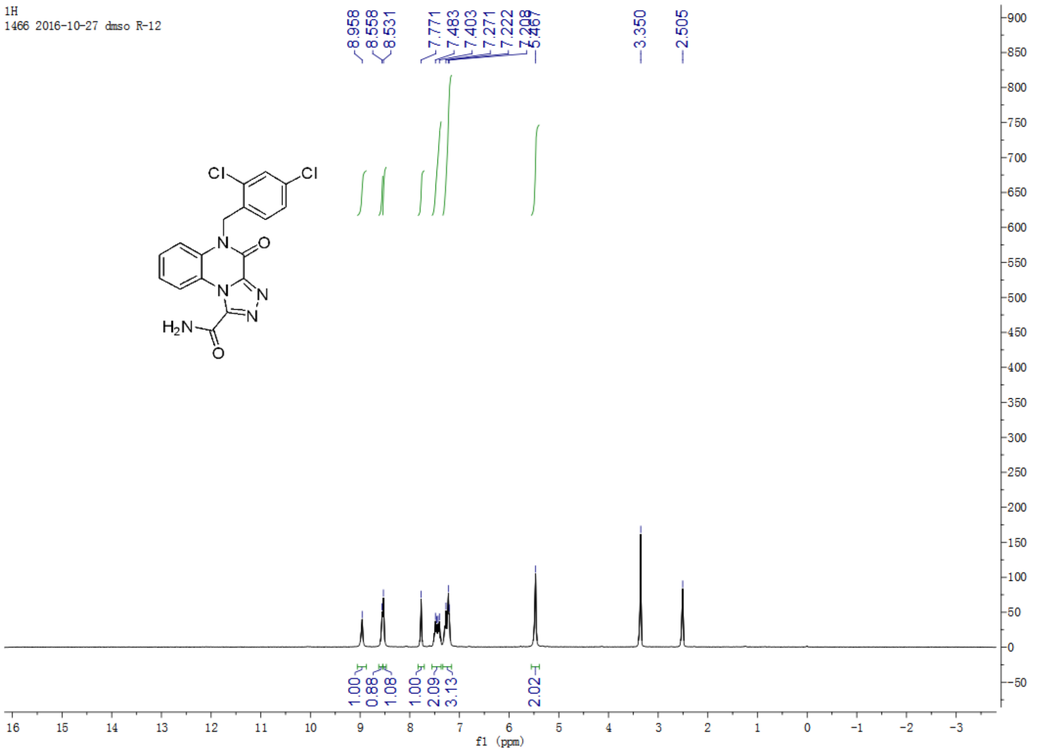

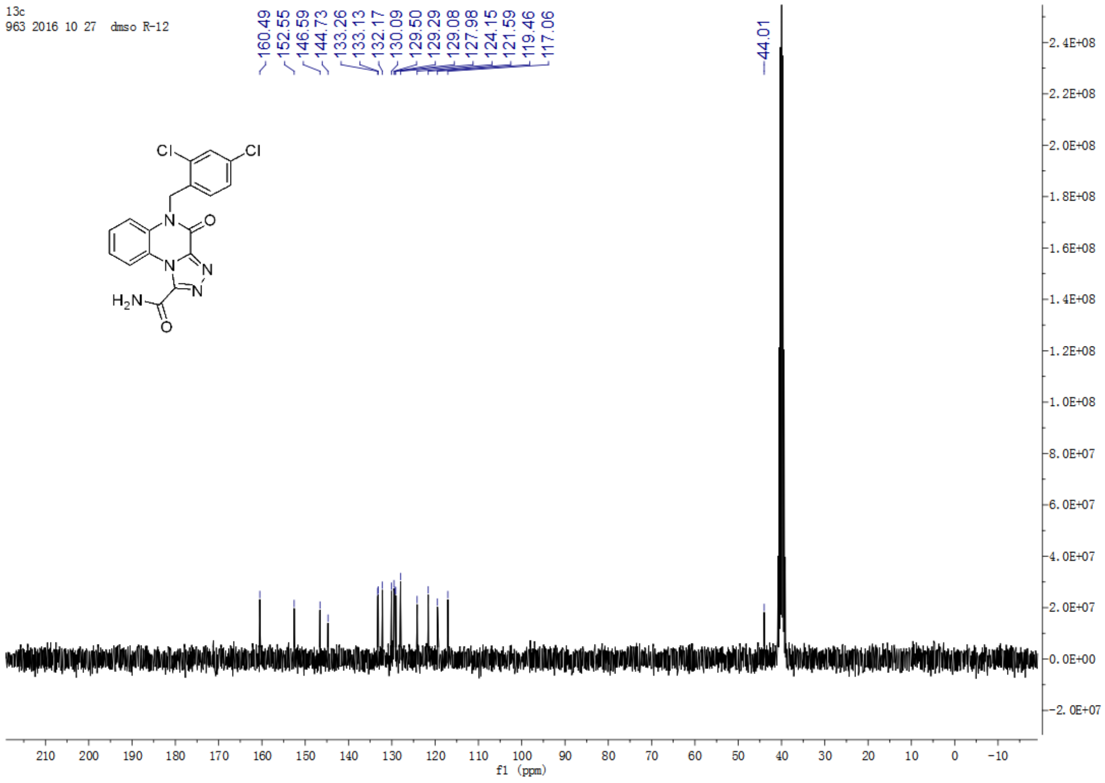

^{1}H NMR and ^{13}C NMR spectra of target compound ***6q***

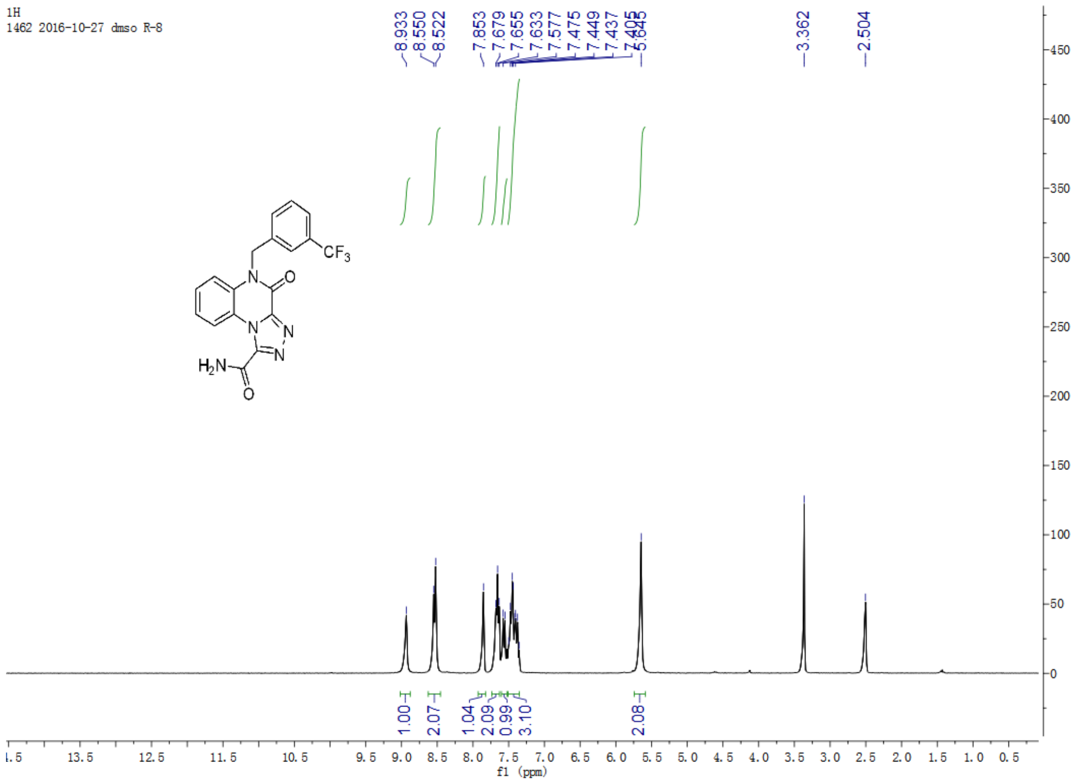

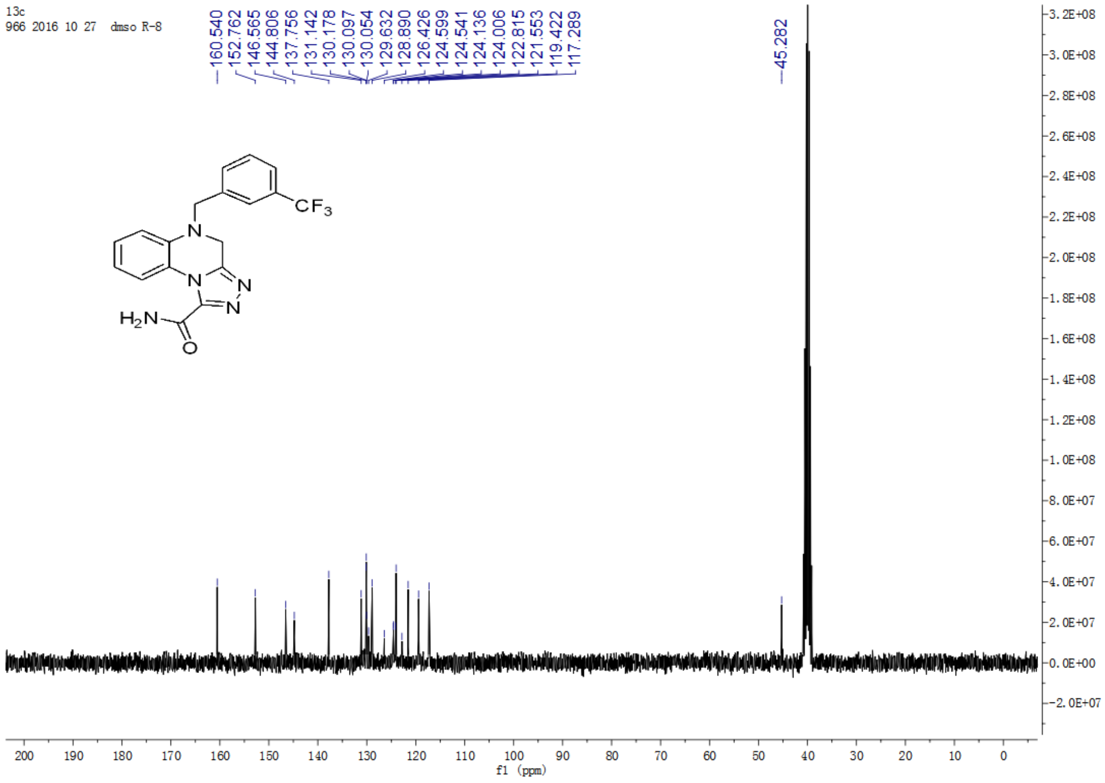

^{1}*H NMR and* ^{13}*C NMR spectra of target compound* ***6r***

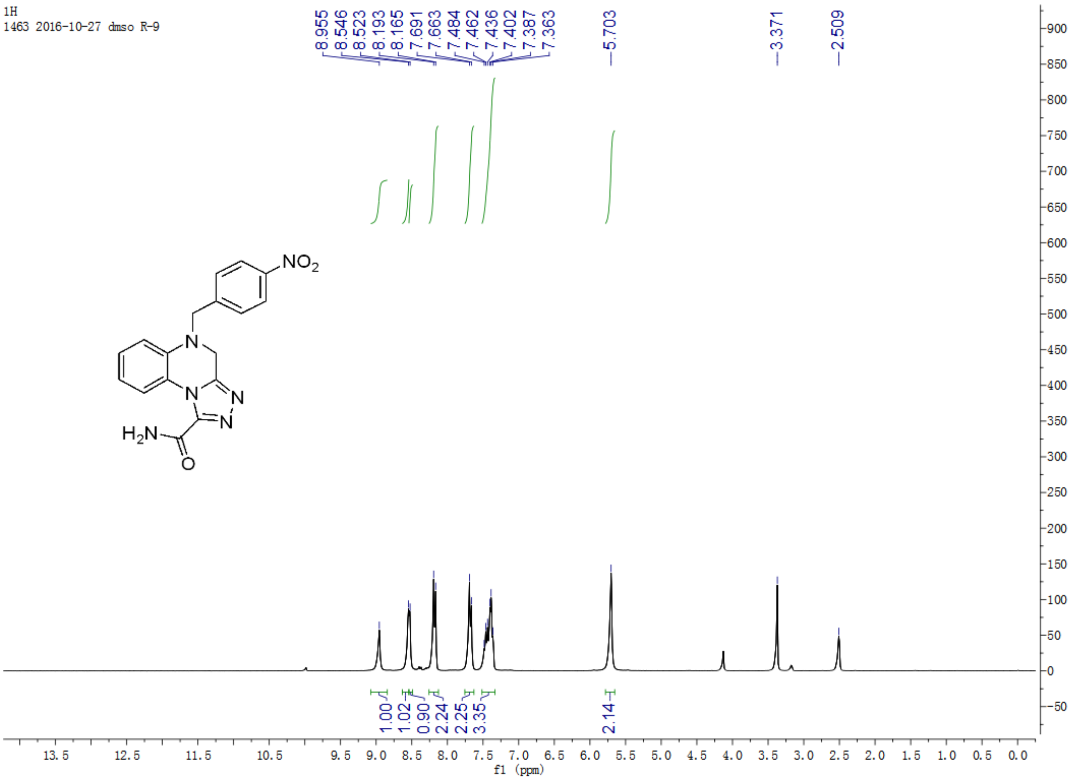

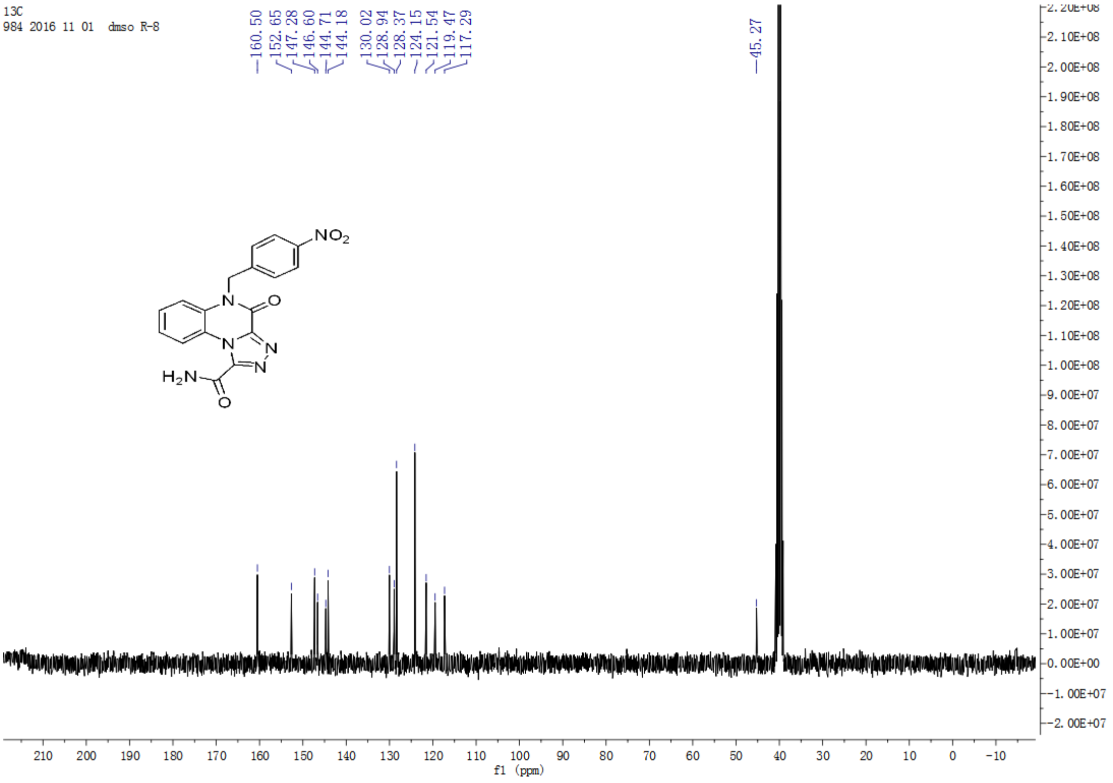

^{1}H NMR and ^{13}C NMR spectra of target compound ***6s***

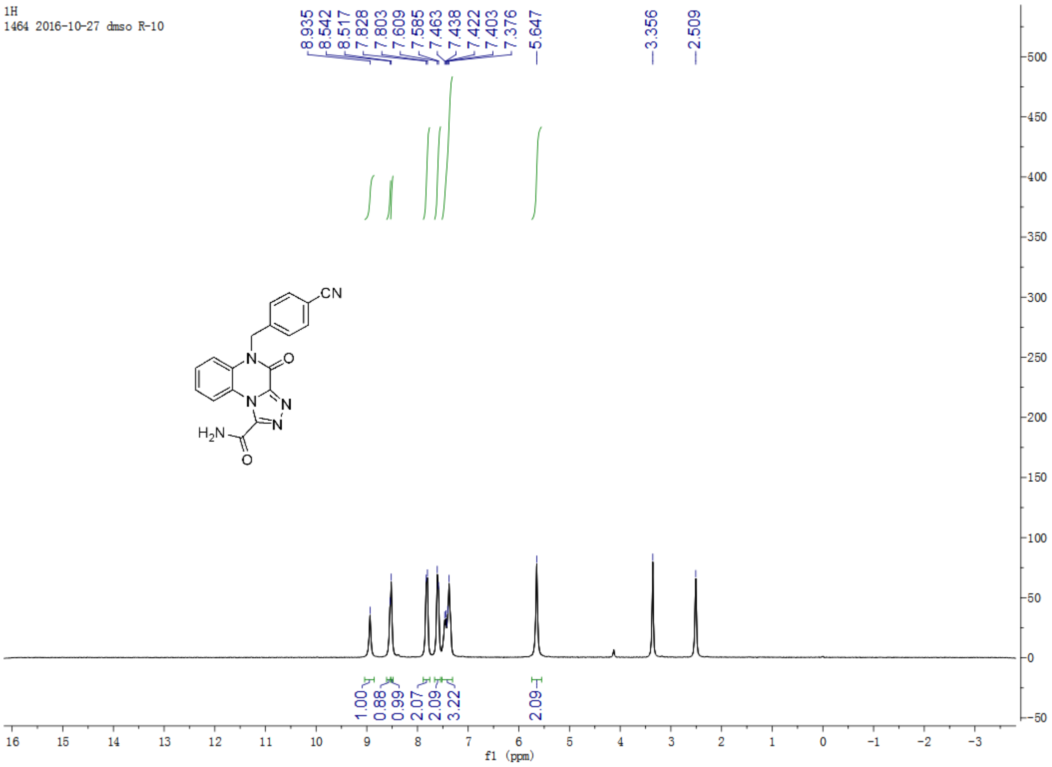

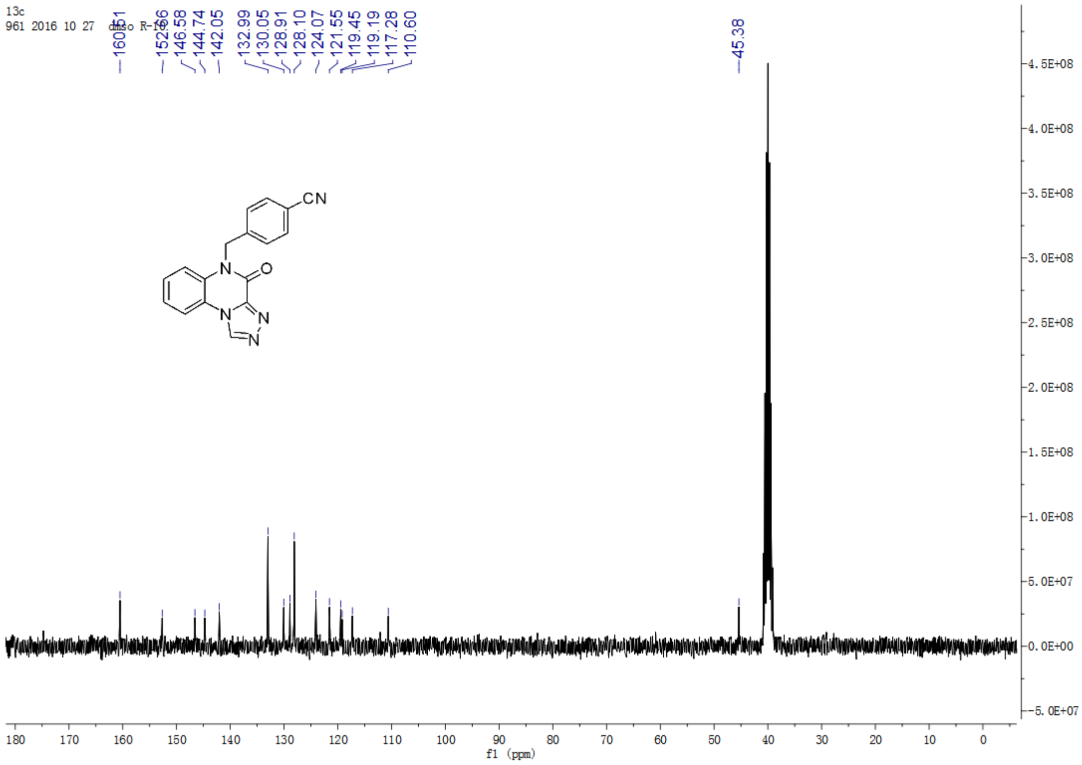

^1H *NMR and* ^{13}C *NMR spectra of target compound* ***6t***

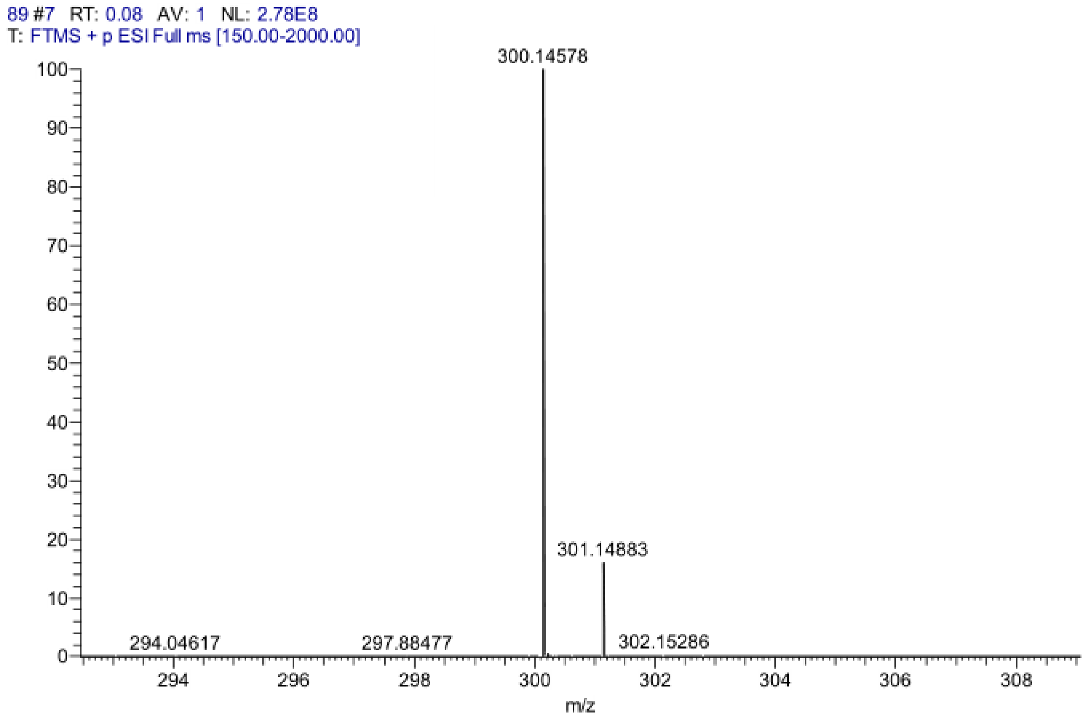

HRMS of target compound ***6b***

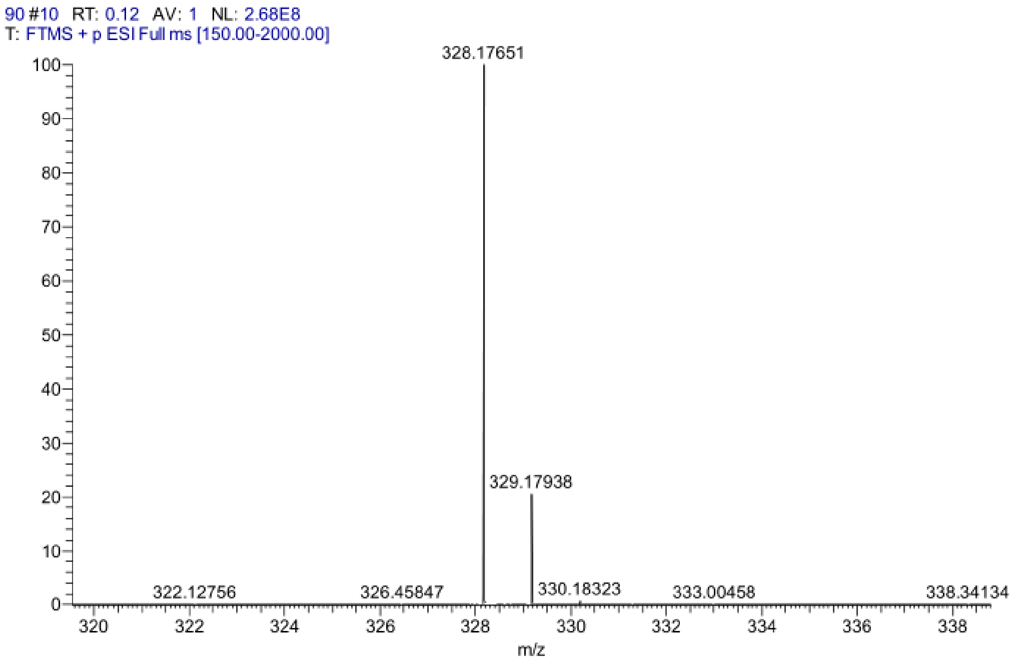

HRMS of target compound ***6d***

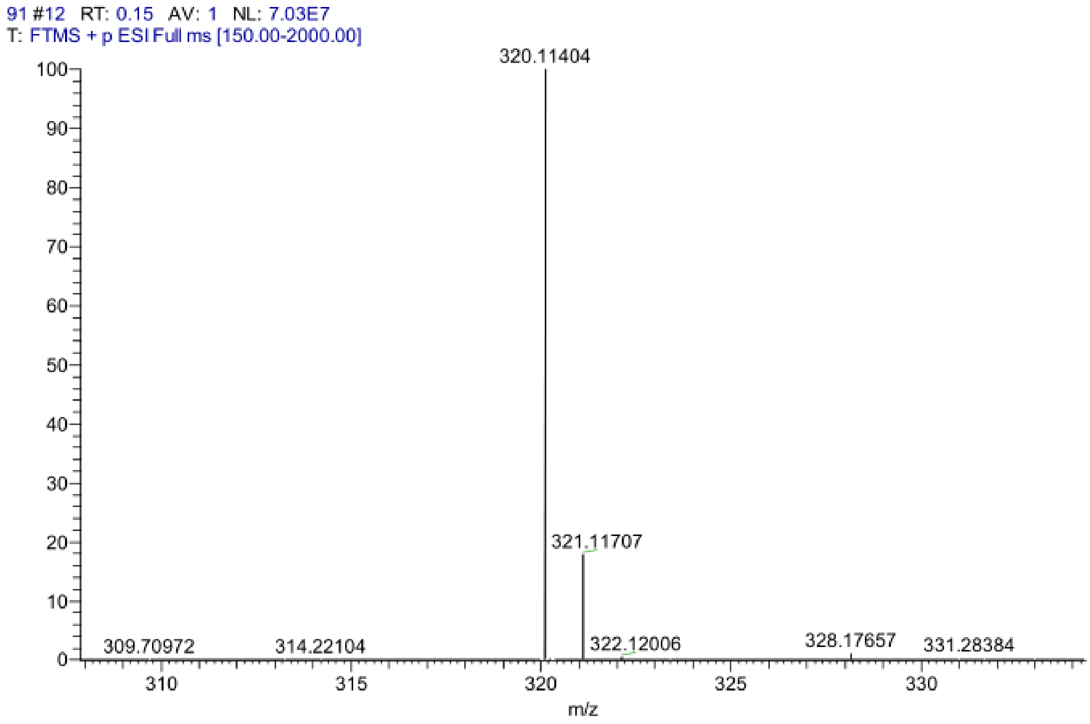

HRMS of target compound ***6g***

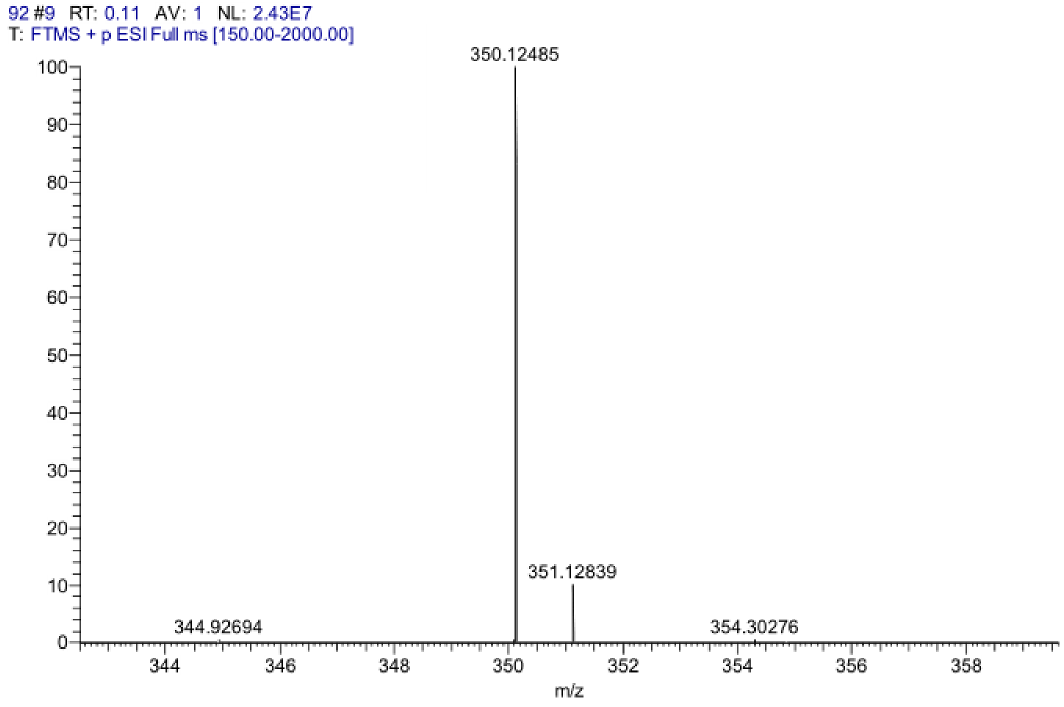

HRMS of target compound ***6n***

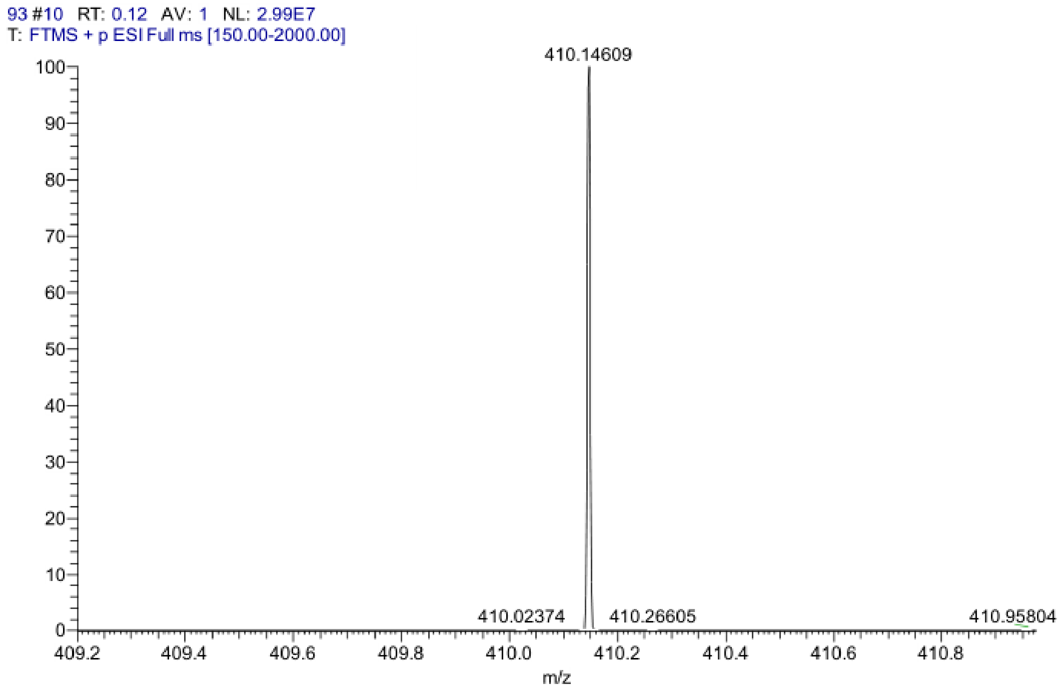

HRMS of target compound ***6p***

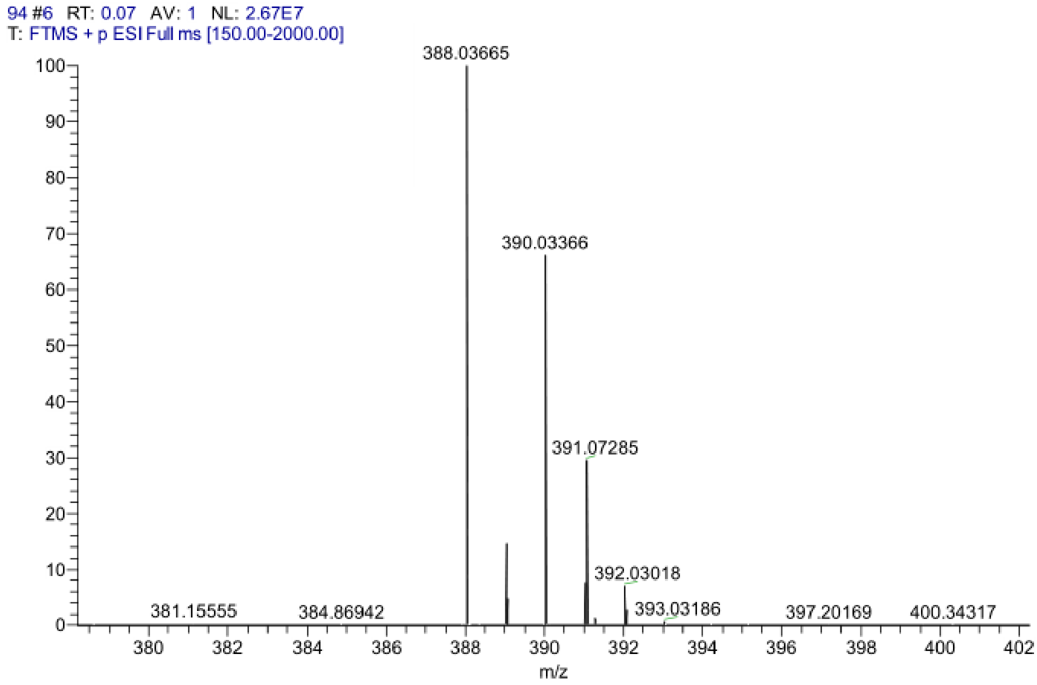

HRMS of target compound ***6q***
